# Dynamic conditioning of porcine kidney grafts with extracellular vesicles derived from urine progenitor cells: A proof‐of‐concept study

**DOI:** 10.1002/ctm2.70095

**Published:** 2024-12-13

**Authors:** Perrine Burdeyron, Sébastien Giraud, Maryne Lepoittevin, Nina Jordan, Sonia Brishoual, Maïté Jacquard, Virginie Ameteau, Nadège Boildieu, Estelle Lemarie, Jonathan Daniel, Frédéric Martins, Nicolas Mélis, Marine Coué, Raphaël Thuillier, Henri Leuvenink, Luc Pellerin, Thierry Hauet, Clara Steichen

**Affiliations:** ^1^ Université de Poitiers INSERM IRMETIST U1313 CHU de Poitiers Service de Biochimie Poitiers France; ^2^ Université de Bordeaux Institut des Sciences Moléculaires UMR‐5255 Talence France; ^3^ Université de Bordeaux INSERM PUMA (Transcriptome) Neurocentre Magendie Bordeaux France; ^4^ Department of Surgery Surgical Research Laboratory University Medical Center Groningen University of Groningen Groningen the Netherlands; ^5^ FHU SUPORT ‘SUrvival oPtimization in ORgan Transplantation’ Poitiers France

**Keywords:** cell therapy, exosomes, extracellular vesicles, kidney preservation, kidney transplantation, machine perfusion, preclinical porcine model, urine progenitor/stem cells

## Abstract

Among strategies to limit ischemia/reperfusion (IR) injuries in transplantation, cell therapy using stem cells to condition/repair transplanted organs appears promising. We hypothesized that using a cell therapy based on extracellular vesicles (EVs) derived from urine progenitor cells (UPCs) during hypothermic and normothermic machine perfusion can prevent IR‐related kidney damage.

We isolated and characterized porcine UPCs and their extracellular vesicles (EVs). Then these were used in an *ex vivo* porcine kidney preservation model. Kidneys were subjected to warm ischemia (32 min) and then preserved by hypothermic machine perfusion (HMP) for 24 h before 5 h of normothermic machine perfusion (NMP). Three groups were performed (*n* = 5–6): Group 1 (G1): HMP/vehicle + NMP/vehicle, Group 2 (G2): HMP/EVs + NMP/vehicle, Group 3 (G3): HMP/EVs + NMP/EVs.

Porcine UPCs were successfully isolated from urine and fully characterized as well as their EVs which were found of expected size/phenotype. EVs injection during HMP alone, NMP alone, or both was feasible and safe and did not impact perfusion parameters. However, cell damage markers (LDH, ASAT) were decreased in G3 compared with G1, and G3 kidneys displayed a preserved tissue integrity with reduced tubular dilatation and inflammation notably. However, renal function indicators such as creatinine clearance measured for 5 h of normothermic perfusion or NGAL perfusate's level were not modified by EVs injection. Regarding perfusate analysis, metabolomic analyses and cytokine quantification showed an immunomodulation signature in G3 compared with G1 and highlighted potential metabolic targets. In vitro, EVs as well as perfusates from G3 partially recovered endothelial cell metabolic activity after hypoxia. Finally, RNA‐seq performed on kidney biopsies showed different profiles between G1 and G3 with regulation of potential IR targets of EVs therapy.

We showed the feasibility/efficacy of UPC‐EVs for hypothermic/normothermic kidney conditioning before transplantation, paving the way for combining machine perfusion with EVs‐based cell therapy for organ conditioning.

**Highlights:**

· UPCs from porcine urine can be used to generate a cell therapy product based on extracellular vesicles (pUPC‐EVs).
· pUPC‐EVs injection during HMP and NMP decreases cell damage markers and has an immunomodulatory effect.
· pUPC‐EVs‐treated kidneys have distinct biochemical, metabolic, and transcriptomic profiles highlighting targets of interest.
· Our results pave the way for combining machine perfusion with EV‐based cell therapy for kidney conditioning.

## Graphical abstract

We show the proof of concept in a preclinical model of the feasibility and efficacy of adding a cell therapy product based on urine stem cell‐derived extracellular vesiclesduring ex vivo kidney dynamic preservation to improve its condition; paving the way for combining machine perfusion with cell therapy for organ conditioning.

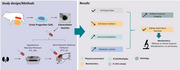

## 1 BACKGROUND

Ischemia‐reperfusion (IR) injuries (IRI), unavoidable in transplantation, is a pathophysiological situation inducing important injury, correlating with graft dysfunction and rejection.^1^ Concomitantly, the organ shortage leads to increasing use of extended criteria donors and donors deceased after circulatory death (DCD) whose organs are very sensitive to IRI. In order to improve these organs’ outcomes, strategies using hypothermic machine perfusion (HMP) before transplantation have been implemented^2^ whereas cell therapies based on stem cells are developing.^3^ Mesenchymal stem/stromal cells (MSCs) partially protect from renal IRI in preclinical/clinical studies.^4^, ^5^, ^6^ Cell therapy has mostly been performed during transplantation anastomosis or immediately post‐transplant.^7^ Considering an earlier stage to administer cell therapy may be interesting and machine perfusion (MP) appears as an optimal time window for extracorporeal organ conditioning before kidney transplantation (KTx).^8^, ^9^, ^10^, ^11^ Compared with static cold storage, HMP increases kidney microcirculation integrity^12^ which may enhance potential cell therapy effect. Gregorini et al.^13^ showed that MSC‐derived extracellular vesicles protect rat kidneys from ischemic injury. HMP can be followed by 1−24 h of normothermic machine perfusion (NMP) to enhance organ condition.^14^, ^15^, ^16^, ^17^ Using NMP to mimic early reperfusion, feasibility studies showed that (1) MSC survival under NMP conditions was satisfying^18^, ^19^ but decreased rapidly^20^; (2) MSC administration during 7 h of NMP lowered porcine kidney injury markers and released immunomodulatory cytokines^20^; (3) 24 h kidney normothermic perfusion with MSCs enhanced renal regeneration.^21^ Feasibility was demonstrated on human kidneys subjected to 7 h of NMP using multipotent adult progenitor cells.^11^ Going one step further, Lohmann et al.^22^ transplanted MSC‐conditioned porcine kidneys but no short‐term beneficial effects were shown.

Extracellular vesicles (EVs) contribute to cell‐cell and cell‐environment communication via their content rich in miRNA, mRNA, and proteins.^23^ They are used as cell therapy product^24^ for their anti‐apoptotic, pro‐proliferative, and pro‐angiogenic effects.^25^, ^26^, ^27^ Regarding organ transplantation, some rodent IR models highlighted the potential of MSC‐derived EVs^28^ which also modulate graft tolerance.^28^, ^29^ MSC‐derived EVs injected during 4 h of HMP in a rat ex vivo kidney perfusion model limit IR kidney injury by protecting the cellular enzymes and cell viability.^13^ In the context of KTx, using cells (or their secretome) from renal origin may be judicious. Urine stem cells (called either urine progenitor cells [UPCs] or kidney progenitor cells) are cells originating from kidney^30^ and isolated from voided urine,^31^ sharing some characteristics with MSCs such as immunomodulatory and pro‐angiogenic properties^32^, ^33^ but their characterization have not led to a consensus yet.^32^ Compared with MSCs, UPCs are isolated with a non‐invasive procedure from an unlimited substrate. Besides differentiation capacity into adipocytes, osteocytes, and chondrocytes (like MSCs), their differentiation into endothelial and epithelial cells has been described.^34^ Moreover, UPCs can be differentiated into renal cells such as tubular epithelial cells or podocytes.^35^ Arcolino et al.^36^ used expanded kidney stem/progenitor cells from preterm neonate urine during NMP of human discarded kidneys showing the strategy feasibility. Using EVs/exosomes during extracorporeal kidney preservation may be optimal to reduce IRI since we hypothesize that kidney perfusion favours EVs renal biodistribution, thereby avoiding some potential risks associated with systemic administration. There is no study to our knowledge describing the use of UPC‐derived EVs in this context.

Our study aims to evaluate the feasibility, short‐term safety, and efficacy of using EVs derived from porcine UPCs (pUPCs) to limit IRI in a preclinical model of porcine kidney HMP followed by NMP.

## 2 MATERIALS AND METHODS

### 2.1 Experimental design

The objective of the study was to isolate and characterize porcine UPCs as well as their EVs and use them as a cell therapy product in a preclinical porcine model of kidney graft dynamic perfusion.

### 2.2 Experimental groups

Porcine kidneys were collected from female pigs from the Kroon Vlees slaughterhouse in accordance with local ethical rules. In this protocol, the farm pigs (Dutch Landrace pigs, exact age unknown, ranging from 6 months to 1 year old) were slaughtered by bleeding (marking the initiation of warm ischemia) and for each animal, one kidney was used. Porcine kidneys (5–6 kidneys/group) were submitted to 32 min of warm ischemia (this timing was exactly monitored and therefore is equivalent for all kidneys), followed by 24 h of HMP (Lifeport perfusion machine [Organ Recovery Systems]) and finally to 5 h of NMP (home‐made experimental design; *cf* Figure 2 for experimental design and Supporting Information Material and Methods). Group 1 was injected with PBS‐1X during both HMP and NMP; Group 2 was injected with EVs during HMP only; Group 3 was injected with EVs during both HMP and NMP (Figure 2).

The following outcomes were assessed: perfusion parameters during HMP/NMP, biochemical analysis of HMP/NMP perfusates during the whole procedure (ASAT, ALAT, LDH, Lactate, Creatinine, Na), cytokine analysis of HMP/NMP perfusates (ELISA and MULTIPLEX), metabolomic analysis of HMP/NMP perfusates (HPLC‐MS /MS), histological analysis of kidney biopsies and transcriptomics analysis of kidney biopsies (RNAseq).

### 2.3 pUPC isolation and characterization

For pUPC isolation, urine was centrifuged at 400 g for 5 min then the pellet was washed with 1× PBS and centrifuged at 400 g for 5 min. The pellet was resuspended with a specific culture medium for the isolation and culture of UPC named “UPC mix” described in a previous study^31^ seeded in a six‐well plate pre‐coated with  .1% (w/v) porcine gelatin, allowing the selection of adherent pUPCs (porcine UPCs). The culture medium was changed every 3 days until a colony appeared (or more). UPC colonies were pooled and cell passaging was performed using Trypsin‐EDTA for amplification and cryopreservation into a UPC mix containing 10% DMSO. Most characterization assays presented in this study were performed on the pUPC3 cell line between passages 2 and 5. For flow cytometry, pUPCs were cultured until reaching 40−80% of confluency (passages 3 and 6) and then 100 000 cells per labeling were used. Antibodies, references, and dilutions are detailed in Table S1). Briefly, cells were trypsinized, washed, and re‐suspended in flow cytometry buffer (1X PBS,  .5% bovine serum albumin [BSA], 2 mM EDTA). For direct immunolabelling, fluorochrome‐conjugated antibodies were added to 100 µL of cell suspension in flow cytometry buffer and incubated for 30 min; cells were then washed three times in the buffer. For indirect immunolabelling, pUPCs were incubated with the corresponding primary antibody for 30 min at room temperature in the dark. Then, cells were washed three times with buffer and then centrifuged at 300 g for 10 min. The secondary antibody was added and incubated for 1 h in the dark at 4°C. The pellets were resuspended in 100 µL of buffer and 20 000 cells were analyzed on an Accuri C6 flow cytometer (Becton Dickinson).

For immunofluorescence, pUPCs were cultured until reaching 40% confluency and then washed with 1× PBS and fixed for 20 min at room temperature with 4% paraformaldehyde. After three washes with 1× PBS, cells were saturated with 1× PBS‐3% BSA for 20 min at RT. Primary antibody was added and cells were incubated overnight at 4°C with gentle agitation. Three consecutive washes were performed with 1× PBS–1% BSA–.1% Tween, then a secondary antibody was added (diluted in 1X PBS–1% BSA) and the cells were incubated for 1 h at RT with gentle shaking at 20 rpm. pUPCs were washed with 5 µg/mL DAPI in 1X PBS–1% BSA for 3 min. Then two consecutive washes were performed with 1X PBS–1% BSA–.1% Tween before a final wash with sterile water. A drop of mounting fluid was placed followed by a coverslip and the cells were observed under an Olympus BX41 fluorescence microscope.

For western blotting, cells were cultured until 80% confluency then trypsinized, and washed with 1× PBS–10% FBS. After centrifugation (200 g, 5 min), cell lysis was performed with RIPA lysis buffer (sterile water + RIPA‐1× at a ratio of 1:1, + 1× antiproteases, and 1× phosphostop). After freezing at −80°C, the samples were sonicated for 1 min. Protein concentration was determined using the Bicinchoninic Acid Protein assay kit (Sigma‐Aldrich) and 20 µg of protein was prepared with Laemmli Buffer. Protein denaturation was performed with Dithiothreitol (23.1 mg/mL in Laemmli 4×) and samples were heated at 95°C for 8 min. Protein extracts were deposited in precast stain‐free 4−15% polyacrylamide gels in electrophoresis buffer containing tris‐glycine buffer and sodium dodecyl sulfate (2.5 mM Tris/19.2 glycine/.01% SDS) allowing migration. Proteins were transferred into a PVDF membrane (polyvinylidene fluorophore) for marker SLA‐DR and HLA‐C and a nitrocellulose membrane for marker AQP1. Control of the total protein migration was performed using the Stain Free technology (Chemidoc imaging system, BioRad). The membrane was saturated for 1 h at RT with 5% (w/v) milk in  .1% TBS (Tris Buffer Saline)‐Tween 20 under agitation. The primary antibody diluted in 5% (w/v) milk was added overnight at 4°C. After three washes for 10 min under agitation with  .1% TBS‐Tween 20, the secondary antibody diluted in 5% (w/v) milk was deposited on the membrane for 1 h under agitation at RT. After four more washes of 10 min with  .1% TBS‐tween 20, protein expression was revealed using Chemidoc with the addition of ECL substrate for electrochemiluminescence and analyzed with a ratio to the total amount of protein.

### 2.4 pUPC differentiation and characterization

LLCPK1 porcine epithelial cells were cultured in basal M200 + 3% FBS until reaching 60−70% confluency. The medium was changed and then recovered after 24 h of culture and centrifuged at 400 g for 5 min to remove cell debris; the supernatant was aliquoted and frozen at −20°C (=conditioned medium). Thawed conditioned medium was used for pUPC differentiation (medium change every 2−3 days using a mixture of UPC mix and conditioned medium (1:1 ratio)), adding extemporaneously human epithelial growth factor (hEGF 236‐EG, R&D System) at 30 ng/mL. Cells were treated for 21 days (D21) and compared with pUPCs before differentiation (D0, undifferentiated cells). Western blotting was performed as described above. For hyperosmolarity assays, NaCl and KCl were added to the medium on the last day of cell differentiation to induce a hyperosmolar medium containing 160 mM of Na^+^, 5 mM of K^+^, and 145 mM of Cl^−^). The expression of different genes of interest was studied by qRT‐PCR after 21 days of differentiation. Genes and primer sequences are detailed in Table S2.

Briefly, cells were lysed and frozen at −80°C to optimize cell lysis, before being thawed and ultrasonicated for 5–10 s. RNA extractions were performed using the NucleoSpin RNA Plus XS kit according to the manufacturer's instructions. The amount of nucleic acid in each sample was assessed with NanoDrop 2000. Prior to retrotranscription, 1 µg of RNA from each sample was run on an agarose gel containing Sybr safe diluted 1000‐fold for 30 min to check the integrity of the extracted RNA and to detect potential DNA contamination. Then, 1 µg of RNA was reverse transcribed using Supermix diluted 1:5. Retro‐transcription was performed using the thermal cycler with the following cycles: 5 min at 25°C, 30 min at 42°C, 5 min at 85°C and 4°C to finalize the reaction. DNA samples at 50 ng/µL were stored at −20°C. Then, 2.5 ng/µL of cDNA was amplified in the presence of the sense and anti‐sense primers (at 250 nM of final concentration each) with Syber Green Mix (Syber Green Quanta SuperMix kit) and performed by the “Roto‐Gene Q” thermal cycler was used. Two reference genes were used (*L19* and *CYA62*) and the Ct of interest was normalized by the “fold change” 2^−ΔΔct^ method, analyzing the relative expression to the control.

### 2.5 EVs isolation

pUPC‐derived EVs were isolated by differential ultracentrifugation. First, EVs‐free FBS was prepared using ultrafiltration (Amicon Ultra‐15 filter tubes) after tube washing with water (4000 g, 10 min). 10 mL of FBS was added to each tube before centrifugation at 3000 g for 55 min; providing efficient removal of the EVs (assessed by Nanosight Tracking Analysis, data not shown). The EVs‐free supernatant (FBS EVs‐free) was recovered, stored at −20°C, and used for UPC mix preparation when pUPC was ready for EVs isolation. pUPCs were cultured in their conventional culture media; 24 h before the cells reached 70–80% confluence, they were incubated in an EVs‐free medium. After 24 h of incubation at 37°C, the culture supernatants were gently recovered and centrifuged at 400 g for 5 min at 4°C. Supernatants were centrifuged again at 2000 g for 5 min at 4°C. These two centrifugation steps allow the removal of cell debris and apoptotic bodies. Then, tubes with supernatants were weighted 2 × 2 and placed in the 50.2Ti rotor (Beckman Coulter) for differential ultracentrifugation. Tubes were adjusted for equivalent weight with sterile 1× PBS at 4°C (Thickwall Polycarbonate Tubes, Beckman Coulter, around 20 mL per tube). All centrifugation steps were performed at 4°C with maximal acceleration and deceleration settings. First centrifugation was performed at 17 000 g for 45 min at 4°C to remove debris, then supernatants were collected in new tubes for second centrifugation at 200 000 g for 65 min at 4°C. Supernatants were discarded and the EVs pellets were collected in 1× PBS. A third centrifugation was performed again at 200,000 g for 65 min at 4°C (to wash the EVs pellet). The EVs pellet was resuspended in 50 µL of 1× PBS. Importantly, to ensure reproducibility and to prevent variation within the cell therapy product, at the end of the EVs isolation process, all our EVs pellets were pooled together and aliquoted before storage at −80°C (around 1 aliquot of EVs/12 M of pUPCs).

### 2.6 Characterization of pUPC‐derived EVs

The presence of specific proteins in EVs such as TSG101 and ALIX was analyzed by western blotting. For each protein marker, EVs samples were compared with a cellular extract from pUPCs as well as from porcine endothelial colony‐forming cells (ECFCs) that were present in the lab and used as a positive control of the porcine stem cell population. EVs samples were directly loaded without prior lysis step; the total protein amount was evaluated by microBCA (ThermoFisher Scientific); and 3 µg of sample was loaded per lane. For transmission electronic microscopy (TEM), samples of pECFC‐ and pUPC‐derived EVs were thawed and analyzed by electron microscopy (JEM 1010, JEOL). Briefly, negative staining was performed on one drop for each sample: the drop was placed on a thin coverslip for 1 min to deposit the EVs on it. The slide was then placed in 7% (v/v) uranyl acetate for 3 min, rinsed with water, and analyzed by TEM. For scanning electronic microscopy (SEM), EVs were thawed and deposited in nitrogen to perform cryofracture and then placed at −80°C for 7 min for a liquid sublimation step. The samples were then metallized with a 3 mm layer of platinum to increase the image resolution using the Leica EM ACE600. Then, samples were analyzed with the FEI scanning microscope. TEM and SEM analyses were performed on the ImageUp platform of the University of Poitiers. For Nano tracking analysis (NTA), 50 µL of the EVs production was sent in dry ice to the NanoMultiPhot platform (ISM, UMR 5255) at the University of Bordeaux, allowing qualitative and quantitative analysis of EVs via the detection of the Brownian motion of the particles present in the solution. Highly diluted samples (1/100 in PBS buffer) were imaged at 25°C on NanoSight400 (Malvern) using a 405 nm laser with a sCMOS camera. The experiments were repeated 5 times with different concentrations to ensure no dependence of the hydrated diameter with concentration.

### 2.7 Ex vivo kidney preservation model

#### 2.7.1 HMP

For each animal, whole blood and a kidney (with preserved ureter and blood vessels) were collected as described above, with bleeding marking the beginning of the initiation of the warm schema (WI). Blood (2.5–3 L) was placed in a beaker containing 25 000 IU of heparin and kidneys were prepared for perfusion during WI. After 32 min of WI, kidneys were washed with 500 mL of kidney perfusion solution (KPS‐1) at 4°C then directly perfused in the Lifeport perfusion machine (Organ Recovery Systems) with 1 L of KPS‐1 during 24 h at 4°C (with a maximum pressure of 35 mmHg at of 5°C ± 1°C). Urine was collected and recirculated during the HMP procedure using this commercial setting.

#### 2.7.2 NMP

After 24 h of preservation with HMP, kidneys were perfused for 5 h by NMP at 37°C with pressure at 75 mmHg ± 15 mmHg + oxygen at  .5 mL/h. An initial bolus of Verapamil was administrated 1 min before perfusion (2.5 mg). The solution used in NMP contained autologous blood cells collected from the pig with 2.7% human albumin,  .07% glucose,  .09% sodium bicarbonate,  .67 mg/mL calcium gluconate, 66.15 mg/mL creatinine, 550 µg/mL/111 µg/mL amoxicillin/clavulanic acid,  .0111 IU of insulin, and 3.5 µg/mL of verapamil. Produced urine was collected via ureter catheterization and not recirculated in this system. Urine was collected every hour and perfusate was completed with the corresponding volume of NaCl. In addition, continuous perfusion of Verapamil was performed during the whole procedure (.25 mg/h or  .4 mL/h, diluted in NaCl). pH and glucose levels were also adjusted when needed (pH < 7.3 and glucose < 4 mM).

Porcine UPC (pUPCs)‐EVs were prepared in PBS‐1X vehicle and we used around 6.5 × 10^9^ EVs per injection (in 1 L final, corresponding to 63.18 ng of total protein of EVs/mL), corresponding to the secretion of approximately 12 million of cells. For injection, EVs were suspended in 32 mL of adequate solution (KPS‐1 for HMP or NMP medium) recovered just before injection and the solution was injected (flow rate of 180 mL/h for 10 min, injection timing: 30 min after the beginning of HMP and/or at 1 h after the beginning of NMP).

For pUPC‐EVs injection and group allowance, sequential randomization was performed (no clustering). We ensured that the learning curve was crossed before starting the study.

#### 2.7.3 Functional parameters during preservation

Biochemical analyses were performed at the Biochemistry department of the Poitiers Hospital on an automated ROCHE COBAS system for LDH, ASAT, ALAT, ionogram, bicarbonate, lactate, and creatinine. Blood and urine samples were first centrifuged and aliquoted before analysis. Measurements were performed and normalized to the kidney weight (weighted after NMP) and to the perfusion volume at each sampling time. The glomerular filtration rate (mL/5 h/100 g of the kidney) over the 5 h of NMP infusion was estimated based on creatinine clearance. Perfusate and urine concentration of creatinine was thus determined at each sampling time and then cumulated over the 5 h of infusion to establish the filtration rate. All cumulative values were used to calculate the creatinine clearance over the total infusion time using Equation (1):

(1) $$\begin{array}{ccc} & & \mathrm{GFR}\;\left(\mathrm{mL}/5\;h/100\;g\right)=\\ & & \;\frac{\left[\frac{\;\mathrm{Urine}\;\mathrm{creatinine}\;\left(5\;h\right)*\;\mathrm{Urine}\;\mathrm{cumulative}\;\mathrm{volume}\;\left(5\;h\right)}{\mathrm{Perfusate}\;\mathrm{creatinine}\;\left(5\;h\right)}\right]}{\left(\frac{1}{100^{\mathrm{th}}}\right)\mathrm{of}\;\mathrm{renal}\;\mathrm{weight}}. \end{array}$$

Cumulative perfusate and urine sodium concentration over the 5 h infusion was calculated to determine the filtration and excretion rate. Cumulative analysis was favoured because NaCl was added to supplement the lost urine volume.

The percentage of renal sodium excretion at the end of the infusion was determined using the cumulative serum and urine concentrations of sodium and creatinine via Equation (2):

(2) $$\begin{array}{ccc} & & \%\;\mathrm{Sodium}\;\mathrm{excretion}\;=\\ & & \;\frac{\mathrm{Urinary}\;\mathrm{sodium}\;*\;\mathrm{serum}\;\mathrm{creatinine}}{\mathrm{Serum}\;\mathrm{sodium}\;*\;\mathrm{urinary}\;\mathrm{creatinine}}\;\;\times \;100. \end{array}$$

### 2.8 Histological analyses

Biopsy samples from corticomedullary kidneys collected at the end of NMP were fixed with 4% formalin and paraffin‐embedded. Renal histological injuries were evaluated (or scored) using periodic acid Schiff and hematoxylin–eosin Safran colouration(s).

Scores were determined using the following scale, analyzing 10 fields per slide.

For cell detachment and tubular dilatation: 0: normal; 1: lesions < 10%; 2: lesions 11−25%; 3: lesions 26−50%; 4: lesions 51−75%; 5: lesions > 75% (extensive necrosis).

For inflammation: 0: no infiltrate; 1: light localized, 2: light diffuse; 3: intense localized; 4: intense diffuse, and 5: extensive.

For immunohistochemistry, the following antibodies were used (CD10, Ventana 790−4506, and Vimentin (Cell Marque 347 M‐16).

Anatomopathological analyses were carried out by a duo formed by a trained anatomopathologist and a nephrologist expert in the porcine model. Both were blind for the samples/groups. All kidneys included in this study were analyzed, that is, 5−6 per group.

### 2.9 Perfusate content analysis

Various cytokines were analyzed using a MILLIPLEX MAP kit Porcine Cytokine/Chemokine Magnetic Bead Panel 13‐Plex, 96‐well plate assay (PCYTMG‐23K‐13PX; EMD Millipore Corporation): GM‐CSF, IFNγ, IL‐1α, IL‐1β, IL‐1ra, IL‐2, IL‐4, IL‐6, IL‐8, IL‐10, IL‐12, IL‐18, and TNF‐α. We used both HMP and NMP perfusates previously centrifuged at 250 g for 5 min and 1100 g for 12 min, respectively. The assay was performed according to the manufacturer's instructions. Briefly, we added 25 µL of buffer + 25 µL of standards, quality controls, or samples + 25 µL of the magnetic beads to each well of the plate. Appropriate controls for background signals were included (corresponding to different perfusion solutions). The plate was sealed, covered with foil, and incubated overnight on a plate shaker at 4°C. Then the plate was washed three times and 50 µL of detection antibody was added to each well. After incubating the plate at room temperature (RT) for 2 h, 50 µL of streptavidin‐phycoerythrin was added per well. After 30 min incubation at RT, the plate was washed three times and 100 µL of sheath fluid was added to each well. Concentrations of markers were measured on a Luminex 200 instrument using Luminex xPONENT software (Luminex Corporation). Quality control values for each marker were consistently within the range indicated by the manufacturer.

ELISA kits to Blood Neutrophil gelatinase‐associated lipocalin (NGAL; Bioporto diagnostics), and Transforming growth factor beta (TGF‐β), angiogenin, Prostaglandine E2, heme oxygenase‐1 (HO‐1), insulin‐like growth factor‐1 (IGF‐1), and vascular endothelial growth factor A (VEGFA) (all kits from Cloud‐Clone Corp) were assessed on HMP and/or NMP perfusates (and urine samples also for NGAL) according to the manufacturer's instructions.

### 2.10 In vitro assays

Porcine aortic endothelial cells (pAOECs) were cultured using their regular medium (M200 basal medium (Gibco) supplement with Endothelial Low Serum Growth Supplement (LSGS, Gibco) and 8% of FBS (Gibco)). When reaching around 80% of confluence, pAOECs were treated with a mix of perfusates and their regular medium (3:1) for 48 h before the XTT assay (Roche Diagnostic)). XTT assay reflects the activity of mitochondrial dehydrogenases. For analysis of the direct impact of EVs, pAOEC were cultured for 48 h and treated with “perfusates” obtained in an ex vivo experiment without kidney but with injection of EVs in the NMP circuit and sampling of perfusate at T0 (before EVs injection), T120 (1 h after EVs injection) and T300 (5 h after EVs injection). The direct impact of EVs within the perfusion solution can be estimated since the absence of a kidney prevents any release of kidney secretome in the system.

For cell metabolic profile analysis, the Seahorse XFp (Agilent Technologies) was used. pAOECs were cultured in specific chamber slides and treated with the 17 perfusates mixed with their regular medium (3:1) for 48 h. Following the manufacturer's instruction to analyze their metabolic profile, cells in XF DMEM with no glucose (pH 7.4) with 1 mM pyruvate, 2 mM glutamine (Agilent, France) were treated as the following: injection of glucose 10 mM final/well at *t* = 15 min, oligomycin 1.5 µM final/well at *t* = 30 min, FCCP 2 µM final/well at *t* = 45 min and rotenone + antimycin A  .5 µM final/well at *t* = 60 min (all compounds were prepared in DMEM with 1 mM pyruvate, 2 mM glutamine, and 10 mM Glucose). The Oxygen Consumption Rate (OCR in pmoles of O_2_/min) was measured using a Seahorse XFp (Agilent). ATP‐linked respiration (mean OCR of the last three baseline data points minus the mean of the three OCR data points after oligomycin addition), basal respiration (mean OCR of the last three baseline data points minus the median of the 3 OCR data points after antimycin A and rotenone addition), maximal respiration (highest of the three OCR values after the addition of FCCP minus the nonmitochondrial respiration), and spare respiratory capacity (highest of the three OCR values after the addition of FCCP minus the mean OCR of the last three baseline data points) were analyzed.

Primary human renal glomerular endothelial cells (HRGEC) were cultured in Medium 200 supplemented with 2% endothelial LSGS and 8% FBS (Invitrogen) in a humidified atmosphere of 5% CO2 at 37°C. Cells were used in passages 4 to 5. Cells were cultured until 90% of confluency and then submitted to cold hypoxia in the cold KPS‐1 solution in a 0% O_2_, 5% CO_2_, and 95% N_2_ atmosphere (Bactal 2 gaz, air‐liquide) in a hermetic chamber at 4°C, for 24 h (mimicked ex vivo cold ischemia period). At the end of this cold hypoxia period, a reoxygenation phase (mimicked reperfusion period) was applied at 37°C with 20% O_2_, 5% CO_2_, and 75% N_2_, replacing the KPS‐1 by Medium 200 plus 2% FBS with or without EVs (63.18 ng/mL) for 5 or 24 h until the XTT test. For indirect assessment of cell survival/proliferation, we used the spectrophotometric XTT assay (Roche).

For endothelial to mesenchymal transition: human umbilical vein endothelial cells (HUVEC) were obtained from pooled donors (Promocell) and used at passage 4. Cells were maintained in endothelial cell growth medium (Promocell) supplemented with 1% penicillin/streptomycin. Cells were treated for 48 and 72 h with human recombinant TGFβ2 and human recombinant IL1β (Peprotech) at a final concentration of 10 ng/mL each. EVs isolated from UPC were added to the culture media at a final concentration of 400 ng/mL concomitantly to the TGFβ2 and IL1β treatment.

### 2.11 Metabolomics

Sample preparation for metabolomics analysis: Perfusates were collected at various times during both hypothermic and normothermic procedures. After collection, the perfusates were kept on ice for up to 2 h and then centrifuged and the supernatant was aliquoted and stored at −80°C until analysis. For analysis, samples (50 µL) were thawed on ice and deproteinized by the addition of methanol (LC‐MS grade) (200 µL) and 20 µL of internal standards mix; the mixture was vortexed, incubated at −20°C (1 h) and centrifuged (30 min, 15 000 g), and the supernatants were collected and stored at −80°C until required for LC‐MS/MS analysis. Quality controls (QC) and conditioning QC samples were also prepared in this way using pooled perfusates.

UPLC‐MS /MS analysis of perfusate samples ^37^ ^(p5)^: untargeted UPLC‐MS/MS data were acquired using a Vanquish HPLC system coupled to an Orbitrap Exploris 120 mass spectrometer (ThermoFisher Scientific). A resolution of 60 000 was used for MS and 16 000 for ddMS (further details provided in Supplementary Information). Chromatographic separation was performed on an ACCUCORE 150 Amide HILIC (2,6 µm, 100 mm × 2.1 mm, ThermoFisher Scientific) and ACQUITY UPLC HSS T3 (1.8 µm, 100 mm × 2.1 mm, Waters) columns, in both positive and negative (ESI+ and ESI−) modes. The column temperature was maintained constant at 35°C in both ionization modes. Elution was performed according to the appropriate conditions of columns as described in the supplementary information. Sample vials were stored at 4°C in the HPLC autosampler, with 10 µL injected for positive and negative ionisation. MS acquisition settings are also described in Supporting Information Material and Methods.

Raw instrument data (.RAW) were exported to Compound Discoverer 3.3 (CD3.3) for deconvolution, alignment, refinement, compound detection, QC correction, and identification with online databases (full workflow and settings are provided in supplementary information). Peak areas from CD3.3 were subsequently exported as a  .csv file. Strict QA criteria (minimum QC coverage of 80% and maximum QC CV of 30%) were applied to the resulting data.

Within this workflow, principal components analysis (PCA) was used to visualize trends, using the R programming environment. Subsequently, OPLS‐DA was performed to highlight variable importance in the projection scores used to identify the features that contributed to the separation of the three groups.

Chemicals & Material: Optima LC/MS grade acetic acid, methanol, acetonitrile, and water, and Pierce LC/MS grade formic acid were purchased from Fisher Scientific. LC/MS grade formic acid, ammonium acetate, and ammonium formate were purchased from Merck. Phree phospholipid removal tubes (1 mL) were purchased from Phenomenex.

Regarding labelled internal standards, succinic acid‐2,3‐13C2, l‐tyrosine‐(phenyl‐3,5‐d2), and d‐glucose‐13C6 were purchased from Merck while l‐lactic acid‐13C3 from Toronto Research Chemicals.

### 2.12 LCMS analysis

All extracts (5 µL injection volume) were analyzed on an Orbitrap Exploris 120 mass spectrometer interfaced with a Vanquish autosampler from ThermoFisher in both positive and negative (ESI+ and ESI−) modes. Samples were analyzed using two chromatographic separations: (1) ACQUITY UPLC HSS T3, 1.8 µm, 100 mm × 2.1 mm column from Waters in both positive and negative ionization mode and, (2) ACCUCORE 150 Amide HILIC, 2,6 µm, 100 mm × 2.1 mm column from ThermoFisher in both positive and negative ionization mode. The column temperature was maintained constant at 35°C in both ionization modes.

For ACCUCORE 150 Amide HILIC, 2.6 µm, 100 mm × 2.1 mm column. The mobile phase was composed of *A* = 5 mM ammonium formate and  .1% formic acid in 95% acetonitrile and 5% water and *B* = 5 mM ammonium formate and  .1% formic acid in 50% acetonitrile and 50% water for positive mode and *A* = 5 mM ammonium acetate and  .1% acetic acid in 95% acetonitrile and 5% water and *B* = 5 mM ammonium acetate and  .1% acetic acid in 50% acetonitrile and 50% water for negative mode. The linear elution gradient from 1% B (0–1 min) to 95% B (9–14 min) was applied in both ionization modes. The initial gradient conditions were restored within 1 min and a 5 min post‐run equilibration was applied to maintain the system reproducibility. The flow rate was 500 mL/min in both ionization modes.

For ACQUITY UPLC HSS T3, 1.8 µm, 100 mm × 2.1 mm column. The mobile phase was composed of *A* = .1% formic acid in 100% water and *B* = .1% formic acid in 95% acetonitrile and 5% water for both modes. The linear elution gradient from 1% B (0–.5 min) to 99% B (10.5–12 min) was applied in both ionization modes. The initial gradient conditions were restored within  .5 min and a 4 min postrun equilibration was applied to maintain the system reproducibility. The flow rate was 300 mL/min in both ionization modes.

In both cases, ESI source conditions were set as follows: ion transfer tube 320°C and vaporizer 400°C, capillary voltage +3200 V in positive mode, and −2700 V in negative mode.

The analysis was performed in the Full MS/ddMS^2^ (data‐dependent MS^2^) mode, under which a Full MS scan event (SE1) was followed by a data‐dependent scan with a fragmentation energy (SE2) applied. Ions of the second scan event then entered the HCD collision cell and were fragmented. The mass spectrometer acquired a Full MS scan at a resolution of 60 000. The automatic gain control (AGC) target (the number of ions to fill C‐Trap) was set to 1.0e^6^ with a maximum injection time (IT) of 100 ms. The scan range of the Full MS scan was from *m/z* 100 to 1500. For the dd‐MS,^2^ the mass resolution was 16 000 FWHM with AGC target at 2.0e,^5^ a maximum IT of 50 ms, and an isolation window of *m/z* 1.5.

Blank extraction samples were injected at the beginning and end of each batch to assess carry‐over and lack of contamination. Isotopically labelled internal standards were added to analytical and QC samples to assess system stability throughout the batch. QC samples, prepared with a standard reference material were also applied to condition the analytical platform, enable reproducibility measurements, and correct for systematic errors.

### 2.13 Transcriptomic analysis

Kidney biopsies were sampled at the end of the NMP procedure and frozen in liquid nitrogen. RNA was extracted using the Nucleospin RNA plus kit (Macherey Nagel) and following the manufacturer's instructions. The RNA concentration was evaluated on NanoDrop (Thermo Fisher). The resulting RNA quality was determined using a Bioanalyzer Instrument (Agilent) and all samples had RNA integrity numbers above 8.1.

RNA sequencing libraries were prepared according to Illumina's protocols using the Illumina Stranded mRNA Prep, Ligation kit (reference no. 20040534). Ten cycles of polymerase chain reaction (PCR) were applied to amplify the libraries. Library quality was assessed using a LabChip GXII Touch Instrument (PerkinElmer) and libraries were quantified by qPCR using the NEBNext Library Quant Kit (E7630), on the LightCycler 480 II Instrument (Roche). Sequencing was performed in paired‐end (2×100 bp) on an Illumina NextSeq 2000 sequencer at the PGTB platform (https://pgtb.fr/).

Under Linux environments (MCIA cluster), the reference pig genome was built with STAR v2.7.5a using Sscofa11.1 assembly (fasta file) and gene annotation (gtf file, release 108). The reads were first trimmed for adapters by cuadapt v4.0 and aligned onto the reference pig genome by STAR v2.7.5a. Subsequent informatics processes of the sequenced reads were locally done under R environments.

Primary evaluation of the 17 sample expression profiles was performed by PCA. Differential gene expression analysis was next performed using the R‐interfaced DESeq2 suite following the removal of genes with no detected expression. Genes were declared differentially regulated when their fold change (FC) was >1.5 with *p *< .05. Functional enrichment analysis was made using the g‐profiler web platform (https://biit.cs.ut.ee/gprofiler/gost). For qRT‐PCR experiments, total RNAs from porcine renal tissues were extracted with a NucleoSpin RNA Plus kit (Macherey‐Nagel), and reverse transcription was performed with qScript cDNA Supermix kit (QuantaBio) following the manufacturer's recommendations. Real‐time PCR assays were carried out on a RotorGene‐Q with Quantinova Sybr Green PCR kit (Qiagen) following the manufacturer's recommendations. DNA primers were designed using Primer‐BLAST and OligoAnalyser (Integrated DNA Technologies Inc). Primer sequences are listed in Table S2.

### 2.14 Statistical analyses

The data set was plotted and analyzed on GraphPad Prism 8.0.2. For most analyses, the area under the curve (AUC) of each group was determined, and then a non‐parametric Kruskal‐Wallis statistical test followed by a Benjamini, Krieger, and Yekutieli multiple comparison test was performed. Statistical difference was determined when *p* < .05. If results were analyzed differently, and statistical tests were mentioned in the figure legends.

## 3 RESULTS

### 3.1 pUPC isolation, characterization, and differentiation

**FIGURE 1:**
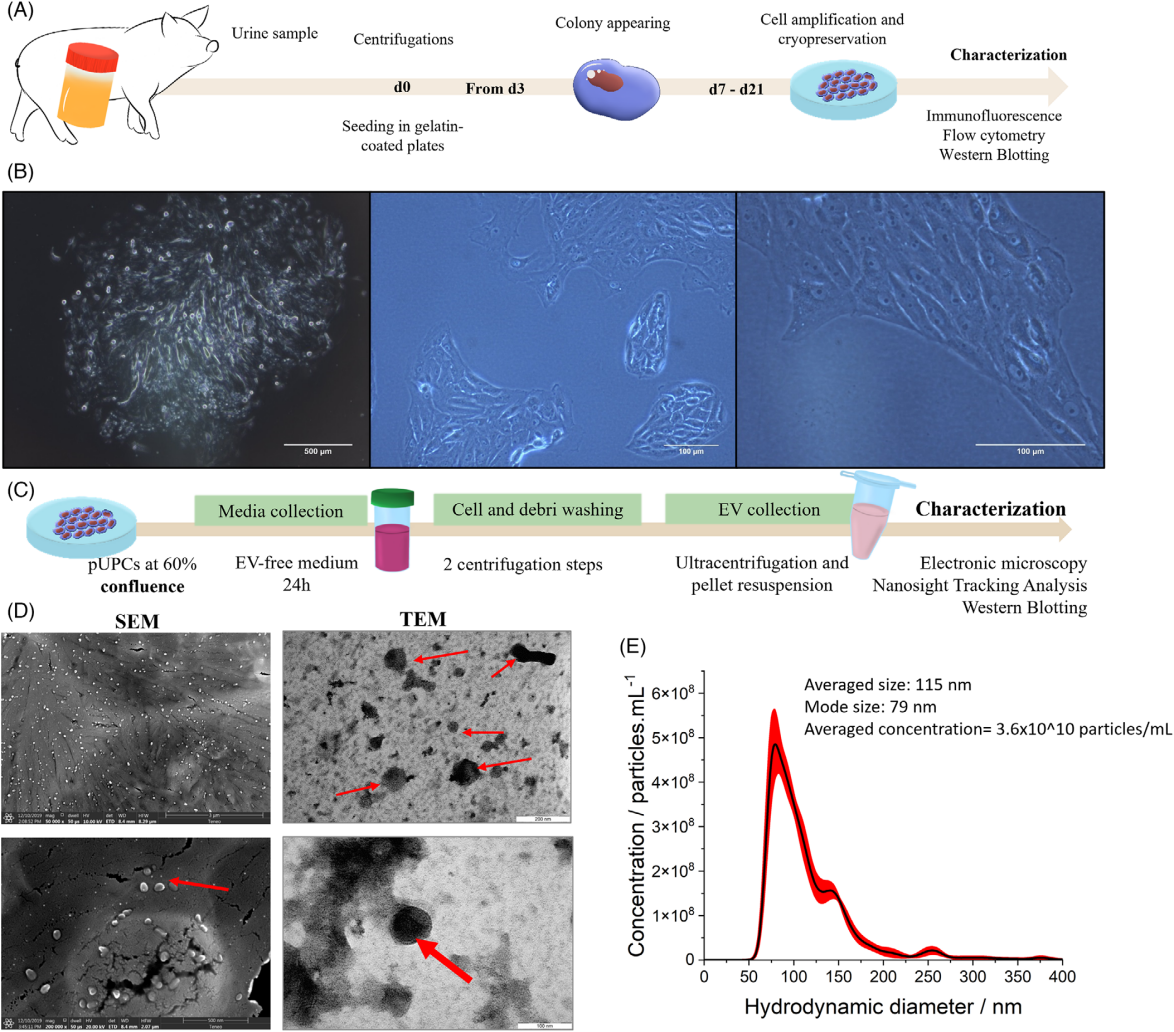
Porcine urine progenitor cells (pUPCs) and EVs isolation/characterization and study design. (A) Isolation of pUPCs from a porcine urine sample. (B) pUPC morphology (left picture: 40×; right picture: 100×). (C) Isolation of EVs from porcine urine progenitor cells. (D) EVs visualization using scanning electronic microscopy (left pictures) and transmission electronic microscopy (right pictures). (E) EVs analysis using nanosight tracking analysis (black line: averaged data; red shape: standard deviation). Averaged size (mean size of all nanoparticles population (full sample)) = 115 nm. Mode size (mean size of the more concentrated nanoparticle population) = 79 nm; averaged concentration = 3.6 × 10^10^ ± .5 × 10^10^ particles/mL.

pUPCs were isolated from pig urine by adapting previously published protocol.^31^ (Figure 1A and material and methods section). pUPCs are adherent cells (Figure 1B) sharing some markers with MSCs such as positive for CD44 and CD90 (Figure S1 for characterization summary and detailed analyses) and negative for CD31 (endothelial) and CD45 (hematopoietic marker). pUPCs express SSEA4 (pluripotent stem cells), CD133 (renal progenitor cells, non‐specific), AQP1, MHC‐I but not MHC‐II. Overall, the porcine UPC profile is very similar to that of human UPCs. To assess pUPC differentiation capacity, we used a conditioned medium from porcine tubular epithelial cells supplemented with epithelial growth factor (30 ng/mL) for 21 days. pUPCs underwent morphological changes (Figure S2A) (confluent monolayer of « pentagonal‐shape » cells) and compared with Day 0 (undifferentiated cells), differentiated UPCs displayed higher gene expression of the epithelial marker E‐cadherin (median fold change: 17.4) and Aquaporin 3 (median fold change: 1520.5; Figure S2B). VIMENTIN, alpha‐SMA, and VCAM1 were not substantially modified. Finally, we analyzed the AQP‐1 protein expression (highly expressed in the proximal tubule). Concomitant with increased AQP1 protein expression at Day 21, we found that the ratio of some larger bands, which may correspond to more mature forms of the protein, was modified after differentiation^38^, ^39^ (Figure S2C).

### 3.2 Isolation of extracellular vesicles from pUPC‐conditioned medium and EVs characterization

EVs were isolated from proliferating pUPCs by differential ultracentrifugation (Figure 1C). Using scanning and transmission electron microscopy, we observed a homogenous population of EVs (average diameter: 80–90 nm; Figure 1D); this was confirmed by nanosight tracking analysis (Figure 1E). pUPC‐EVs expressed ALIX and TSG101 proteins (Figure S3).

### 3.3 Safety and efficacy of pUPC‐EVs during HMP

**FIGURE 2:**
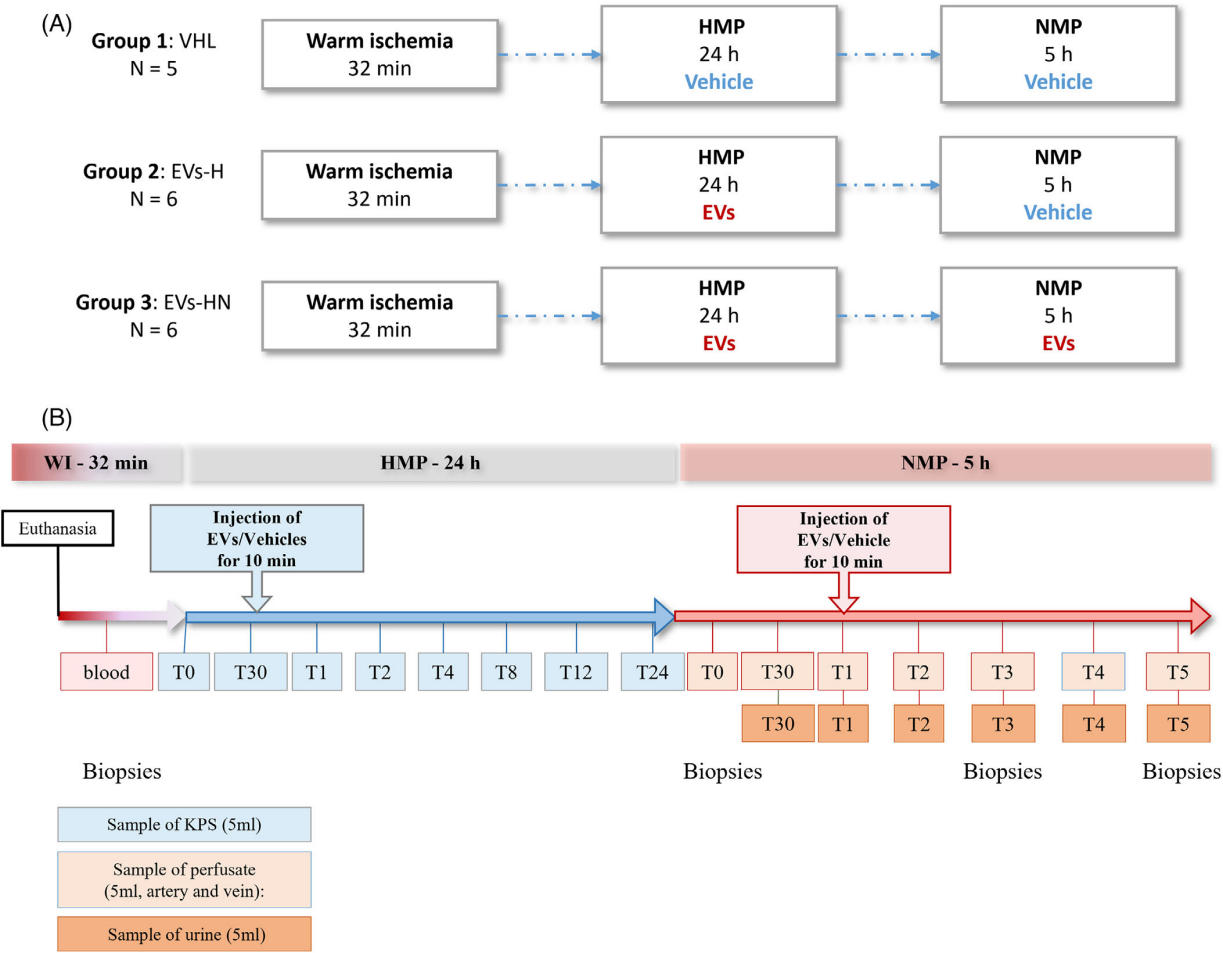
Experimental groups and design. (A) Experimental groups: Group 1 (G1 – Vhl) received only PBS (Vhl) during hypothermic machine perfusion (HMP) and normothermic machine perfusion (NMP) (*n* = 5 kidneys for G1); Group 2 (G2 – EVs‐H) received pUPC‐EVs during HMP and PBS during NMP (*n* = 6 kidneys for G2): Group 3 (G3 – EVs‐HN) received pUPC‐EVs during both HMP and NMP (*n* = 6 kidneys for G3). (B) Experimental design and samplings at different timings (T for timing) (detailed Material and Methods for details).

To assess the safety and efficacy of pUPC‐EVs for ex vivo kidney conditioning, we used an ex vivo porcine kidney preservation model described in Figure 2 (and Material and Methods section), performing three groups (5–6 kidneys/group).

**FIGURE 3:**
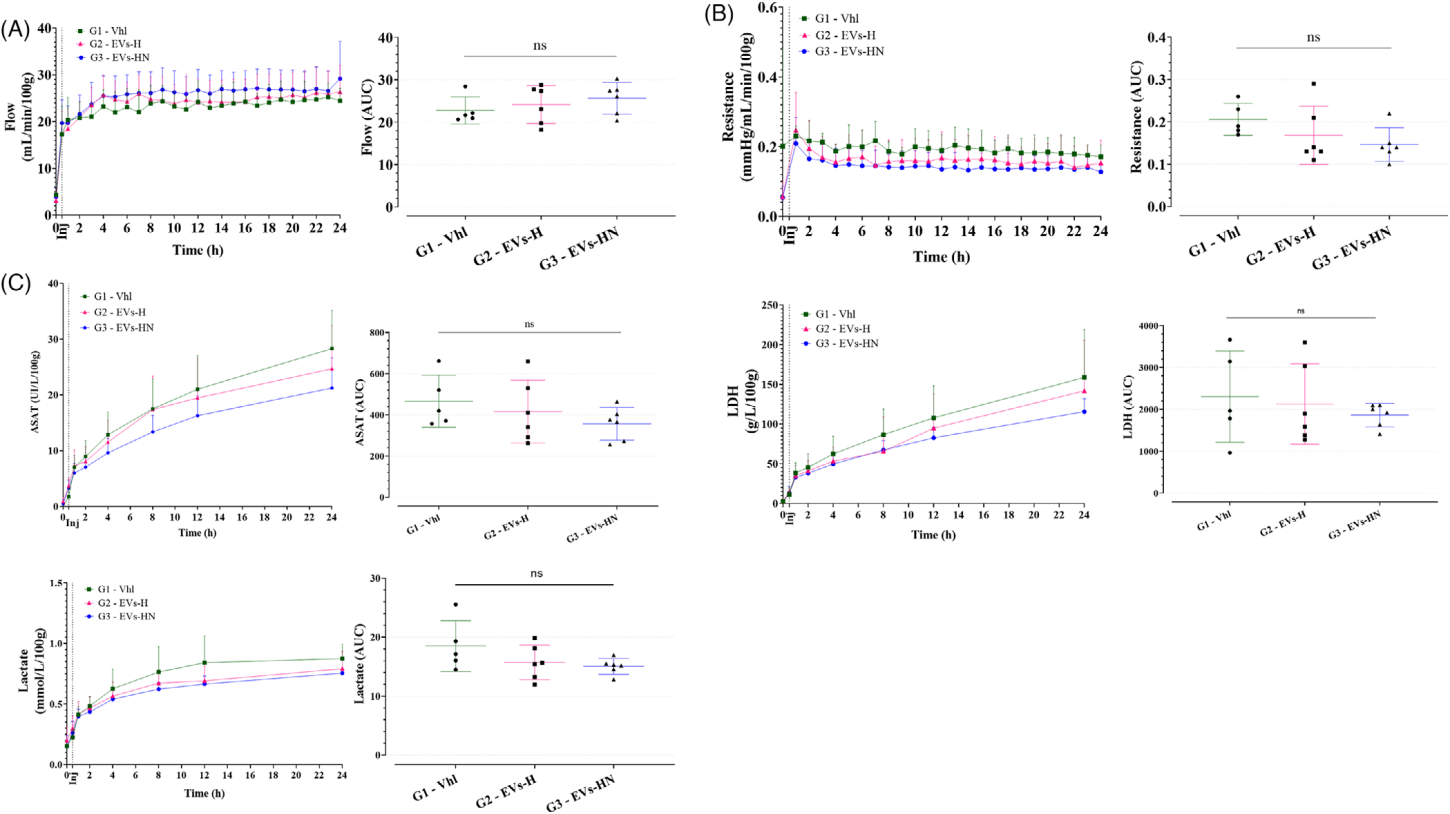
Safety and efficacy of pUPC‐EVs during 24 h of HMP. (A) Perfusion flow expressed in ml/min/100 g of kidney weight during the 24 h of HMP; time is represented in h; data were monitored continuously on the LifePort software; left panel: kinetics; right panel: AUC analysis. (B) Kidney resistance in mmHg/mL/min/100 g of kidney weight during the 24 h of HMP; time is represented in hours; data were monitored continuously on the LifePort software; left panel: kinetics; right panel: AUC analysis. (C) Concentration of cell damage markers in the HMP perfusates: ASAT, LDH, and lactate; left panel: kinetics; right panel: AUC analysis. (ns if *p* > .05, * if *p* ≤.05, ** if *p* ≤  .01, *** if *p* ≤  .001.).

pUPC‐EVs were injected in the HMP circuit 30 min after the initiation of HMP initiation (needed time for stabilization of perfusion parameters), for both Group 2 (G2–EVs‐H) and Group 3 (G3–EVs‐HN). Compared with Group 1 (G1–Vhl), pUPC‐EVs did not affect perfusion parameters (perfusion flow and kidney resistance) which stabilized around 2 h of perfusion (Figure 3A,B). ASAT, LDH, and lactate gradually accumulate during the 24 h of HMP. ASAT and LDH levels (AUC analysis) did not significantly vary among the groups (Figure 3C). pUPC‐EVs injected in G2 and G3 tended to decrease the levels of lactate (*P* = .090.0 for G3 vs. G1). Thus, pUPC‐EVs injection at HMP in our system appeared feasible and safe since no adverse events linked to the injection were reported; however, no beneficial effect of pUPC‐EVs was highlighted during the 24 h of HMP.

### 3.4 Safety and efficacy of pUPC‐EVs during NMP

**FIGURE 4:**
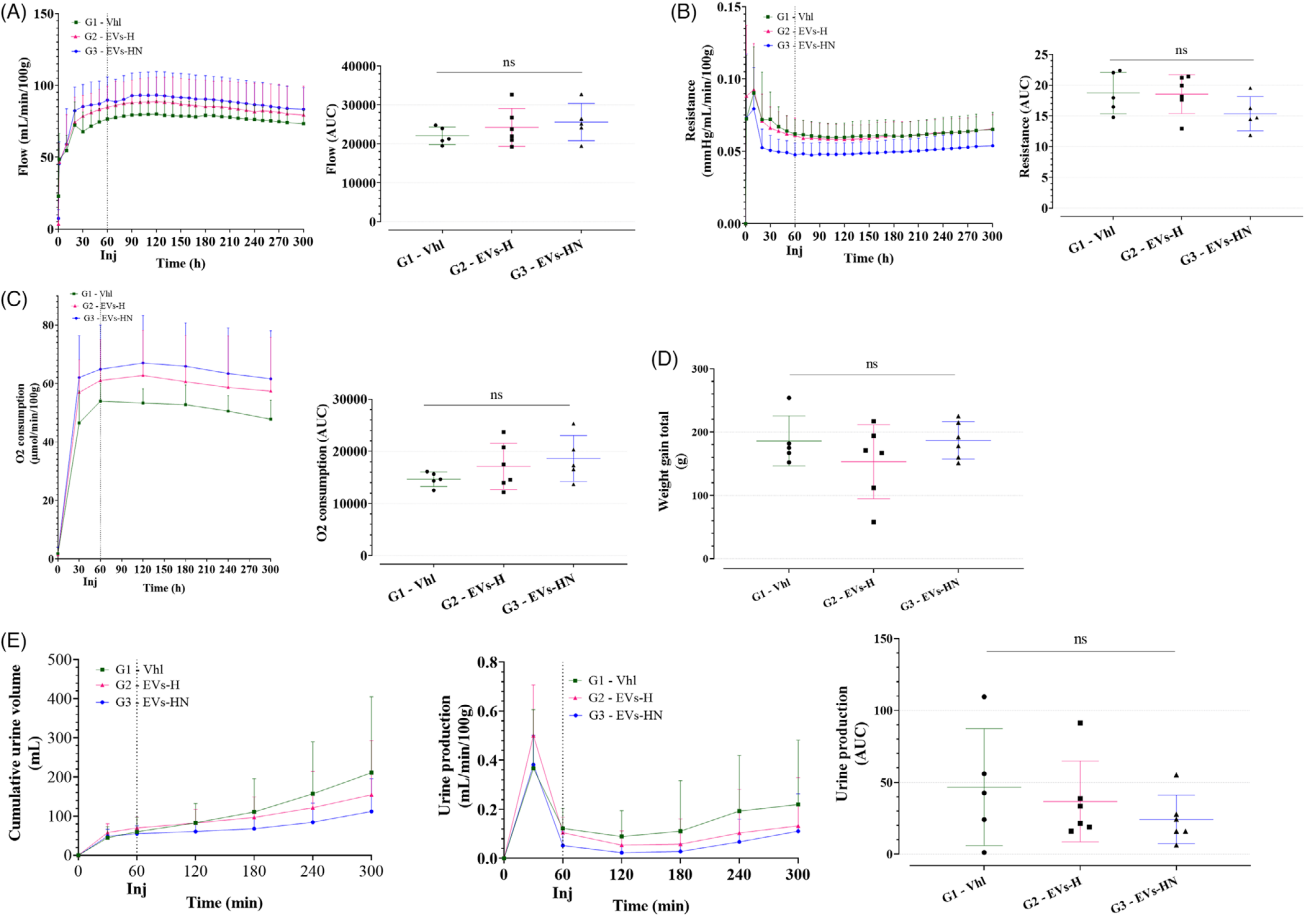
Safety and efficacy of pUPC‐EVs during 5 h of NMP (part 1). (A) Perfusion flow expressed in ml/min/100 g of kidney weight during the 5 h of NMP; time is represented in minutes; left panel: kinetics; right panel: Area under the curve (AUC) analysis. (B) Kidney resistance in mmHg/mL/min/100 g of kidney weight during the 5 h of NMP; time is represented in minutes; left panel: kinetics; right panel: AUC analysis. (C) Oxygen consumption in µmol/min/100 g of kidney weight during the 5 h of NMP; time is represented in minutes; left panel: kinetics; right panel: AUC analysis. (D) Kidney weight gain during the whole procedure (HMP + NMP) in grams. (A) Urine production; left panel: cumulative urine volume (mL); middle panel; urine production in ml/min/100 g of kidney weight; right panel: AUC analysis. (ns if *p* > .05, * if *p* ≤  .05, ** if *p* ≤  .01 and *** if *p* ≤  .001.).

**FIGURE 5:**
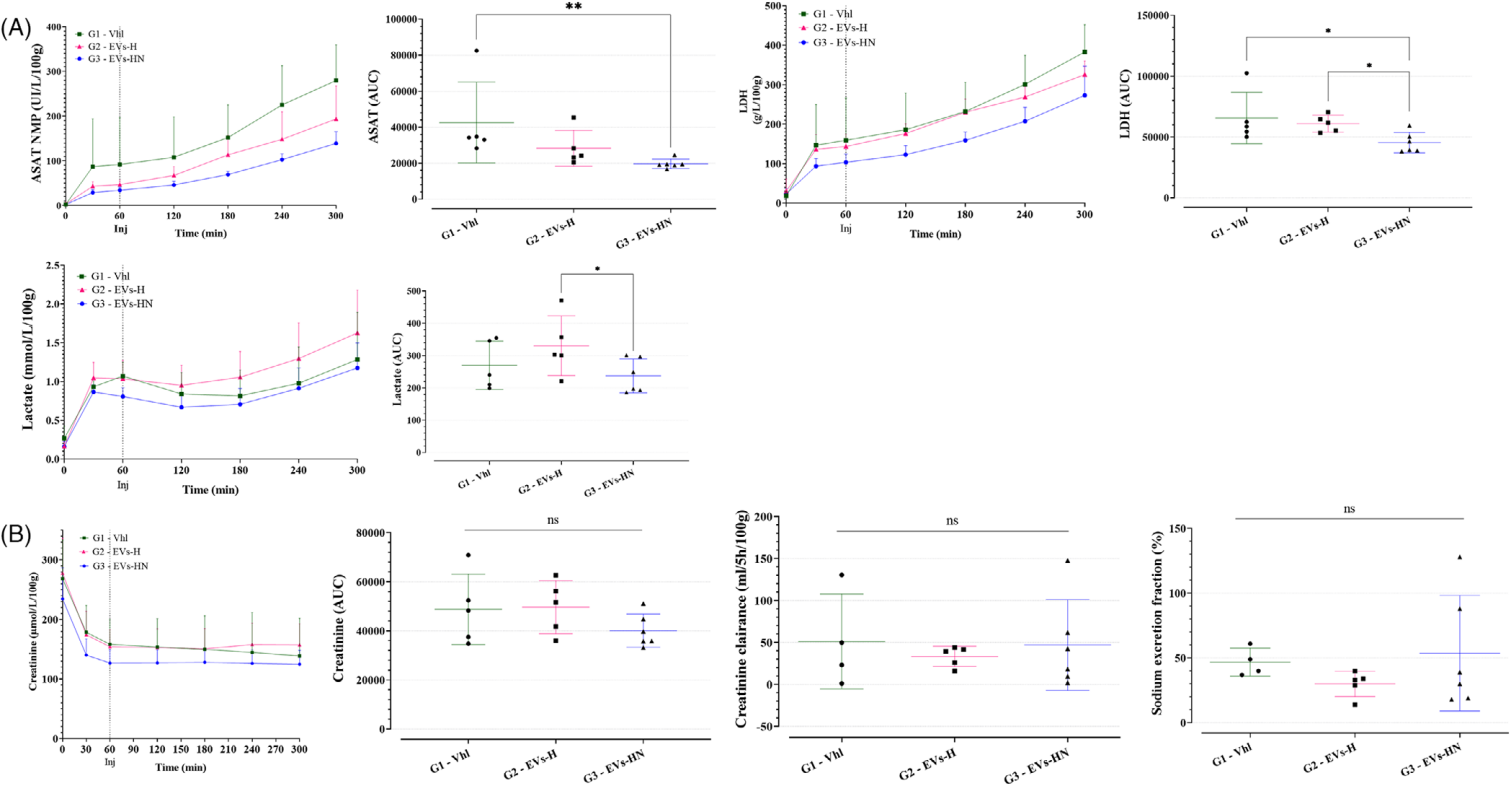
Safety and efficacy of pUPC‐EVs during 5 h of NMP (part 2). (A) Concentration of cell damage markers in NMP perfusates: aspartate aminotransferase (ASAT), lactate dehydrogenase (LDH), and lactate; left panel: kinetics; right panel: AUC analysis. (B) Renal function markers: Creatinine in µmol/L/100 g of kidney weight (left panel: kinetics; right panel: AUC analysis), Creatinine clearance (mL/5 h/100 g of kidney weight), and sodium excretion. (ns if *p* > .05, * if *p* ≤  .05, ** if *p* ≤  .01 and *** if *p* ≤  .001.).

pUPC‐EVs were injected in G3 one hour after the NMP initiation. pUPC‐EVs did not significantly impact perfusion flow, kidney resistance, and oxygen consumption (Figure 4A–C). Kidney weight gain (between HMP initiation and NMP end) was similar within groups (Figure 4D) as well as urine production (Figure 4E). ASAT and LDH were significantly decreased in G3 compared with G1 and Lactate was decreased in G3 compared with G2 but not to G1 (*p* = .05, Figure 5A); of note, G3 kidneys seemed to have lower LDH and Lactate levels at the time of EVs injection. Renal function markers such as perfusate creatinine level, creatinine clearance, and sodium excretion fraction were not modified during the 5 h of NMP by the EVs injection (Figure 5B).

### 3.5 Kidney biopsy histological analysis

**FIGURE 6:**
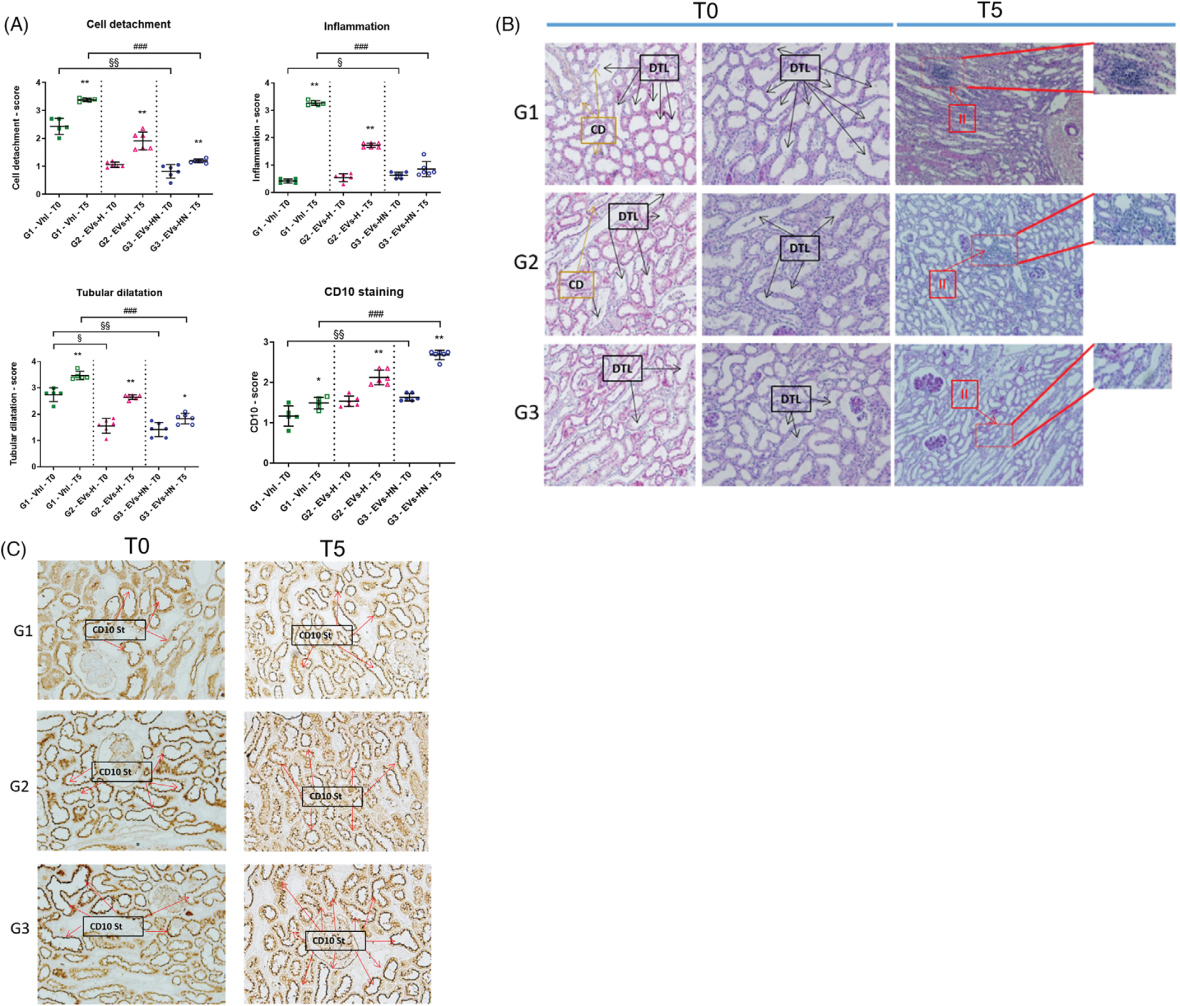
Histological analysis of kidney biopsies: CD, cell detachment; DTL, dilatated tubule; CD10 St: CD10 staining; II, inflammated interstitium. Scores were determined using the following scale, analyzing 10 fields per slide. For cell detachment and tubular dilatation: 0: normal; 1: lesions <10%; 2: lesions 11−25%; 3: lesions 26−50%; 4: lesions 51−75%; 5: lesions > 75% (extensive necrosis). For inflammation: 0: no infiltrate; 1: light localized, 2 light diffuse; 3: intense localized; 4: intense diffuse and 5: extensive. For CD10 staining: number of positive cells per field (10 fields). Meaning for statistical differences. Within each group, T0 versus T5: *p < .05, **p < .01, ***p < .001 (Mann–Whitney test). Between groups, T0 vs. T0: §*p* < .05, §§*p* < .01, §§§*p* < .001 (Kruskal–Wallis + Dunn's multiple. comparisons test), #*p* < .05, ##p < .01, ###*p* < .001 (Kruskal–Wallis + Dunn's multiple comparison test.

We performed kidney biopsies at the beginning of NMP (T0, also corresponding to the end of the HMP) and at the end of NMP. Firstly, NMP itself induced kidney lesions with evidence of tubular dilatation, cell detachment, and inflammation whatever the group. Our results seemed to show that G3 kidneys displayed the most preserved tissue integrity after 5 h of normothermic perfusion, regarding these criteria (Figure 6). Immunohistochemical staining of CD10 and Vimentin showed that G3 kidneys displayed higher expression of CD10 (intact brush border staining) and Vimentin (Figure 6 and Figure S4).

### 3.6 Analysis of normothermic perfusate composition

**FIGURE 7:**
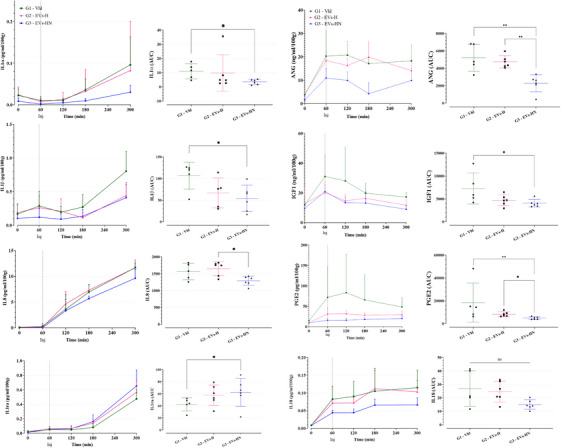
Analysis of normothermic perfusate content: Concentration of cytokines/growth factors in the NMP perfusates: IL1α, IL1β, IL8, IL1ra, ANG, IGF1, PGE2, and IL18. Left panel: kinetics; right panel: AUC analysis. (ns if *p* > .05, * if *p* ≤ .05, ** if *p* ≤ .01 and *** if *p* ≤ .001.).

We investigated whether the HMP and NMP perfusion liquid compositions were affected by the presence of pUPC‐EVs and/or their effect on the renal tissue and its secretion. We quantified proteins of interest in HMP/NMP perfusates collected at different time points (Figure 2B). Over a range of selected targets, only five were detected in HMP perfusates: IL6, IL8, IL12, IL18, and HO1 (Figure S5). No change was detected between groups except for IL18, which significantly decreased in G3 versus G1 and G2 (adjusted *p *= .037 and  .025, respectively, Figure S5). HO1 was detected in G1 only (data not shown). During NMP, some proinflammatory markers were decreased in G3 versus G1 such as IL1α and IL1β whereas the anti‐inflammatory protein IL1RA was increased in G3 versus G1, while IL10 and TGFβ did not vary (Figure 7). NGAL levels did not vary among our groups whereas IL‐18 decreased in G3 versus G1 and G2 almost reaching significance (adjusted *p* = .063 and *p* = .051, respectively; Figure 7 and Figure S6).

### 3.7 Analysis of NMP perfusates impact on endothelial cells in vitro

**FIGURE 8:**
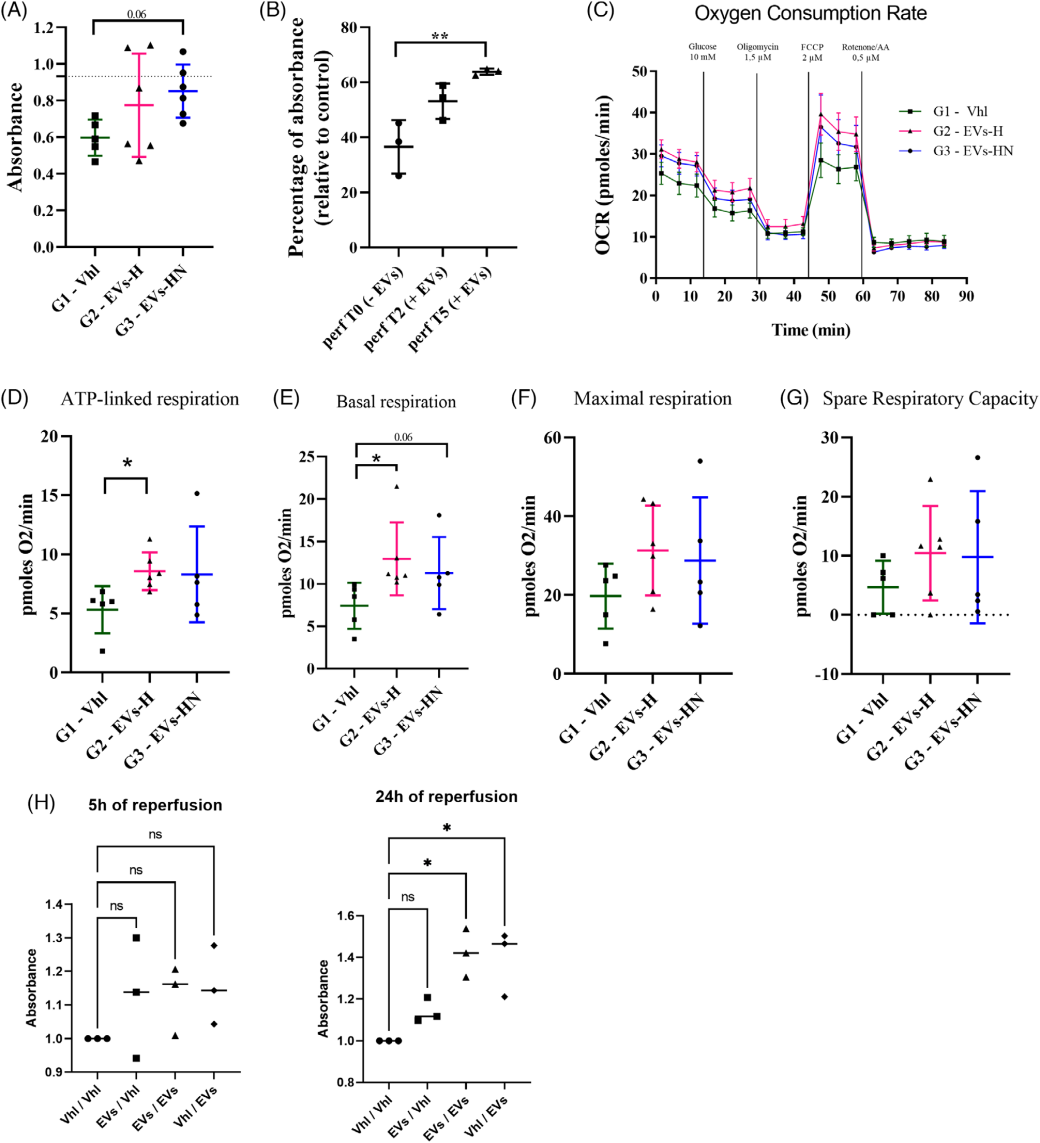
In vitro impact of perfusates and pUPC‐EVs on pAOECs (A–G) and hRGECs (H). (A,B) Mitochondrial activity (absorbance value after XTT assay) of pAOECs (porcine aortic endothelial cells) in basal condition cultured for 48 h with (A) a mix of their regular medium and ex vivo perfusates (diluted perfusates); (B) diluted perfusates from a “blank” NMP procedure when EVs were injected but without kidney on the perfusion circuit. T0: no EVs (before the injection occurring at *t* = 60 min), T2: 2 h of NMP (1 h after EVs injection), T5: 5 h of NMP (4 h after EVs injection). (C–G) Seahorse analysis of pAOECs in basal condition cultured for 48 h with diluted perfusates; data are representative of the metabolic activity from 10 000 cells; (C) Oxygen consumption rate: (D) ATP‐linked respiration; (E) Basal respiration; (F) Maximal respiration; (G) Spare respiration capacity; (H) Mitochondrial activity (absorbance value after XTT assay) of hRGECs (human renal glomerular endothelial cells) in hypoxia‐reoxygenation treated with pUPC‐EVs (or PBS as vehicle, group Vhl/Vhl) either in the hypoxia phase (group EVs/Vhl), during both hypoxia and normothermic reoxygenation (EVs/EVs) or during normothermic reoxygenation only (Vhl/EVs). See the Materials and Methods section for details regarding cell culture protocols and metabolic analyses with the Seahorse analyzer. (ns if *p* > .05, * if *p* ≤ .05, ** if *p* ≤ .01 and *** if *p* ≤ .001.).

To complete our secretome analysis, we used NMP perfusates on pAOECs cultured in normoxia. Cells treated with G3 perfusates tended to have a higher metabolic activity (XTT assay) compared with G1 (*p* = .0601; Figure 8A). To understand if this observation was due to the direct presence of EVs or mediated by the indirect renal release of cytokines/growth (which could be impacted by EVs presence), we performed an extra NMP procedure without porcine kidney on the circuit and gathered perfusate at T0 (before EVs injection), T2 (1 h after injection), and T5. We observed that T5 perfusate increased pAOEC metabolic activity compared with T0 (*p* = .0146), suggesting that at least part of the effect is due directly to EVs (Figure 8B) but it could not be strictly established because EVs presence after 5 h of perfusion was not verified. We then evaluated the metabolic state of pAOECs treated with NMP perfusates using a Seahorse analyzer. We observed that cells respond to our protocol (decrease of OCR following Oligomycin treatment [Figure 8C]); however, ATP‐linked respiration, known to be sensitive to Oligomycin, was higher for G2 than G1 (G2 versus G1: *p* = .04; G3 versus G1: *p* = .41; Figure 8D). Regarding respiration capacity, basal respiration was increased in G2 (and trend for G3; Figure 8E), but in the presence of oxygen and glucose, the maximum respiration capacity and the spare respiratory capacity were not significantly modified by our perfusates (Figure 8F,G).

Finally, we reproduced our ex vivo preservation model in vitro in a human model using primary endothelial cells, the first cells facing EVs in our model. Human renal glomerular endothelial cells were subjected to 24 h of cold anoxia (4°C) in KPS‐1 preservation solution followed by either 5 or 24 h of normothermia with 20% O_2_. Control cells were treated with PBS‐1X in both hypoxia and normoxia (mimicking G1) and some cells were treated either in the hypoxia phase only (mimicking G2) or in both hypoxia and normoxia (mimicking G3) (same concentration as ex vivo). We added a condition where cells were treated only during the normoxia phase. Using the XTT assay, we showed an increased metabolic activity when cells were treated with pUPC‐EVs both in cold hypoxia and normothermic reoxygenation, compared with control (Figure 8H). However, we only observed a significant increase 24 h post‐reoxygenation, whereas no significant change was detected after 5 h of reperfusion. Similar results were observed when cells were kept under normothermia.

Finally, we used TGFβ2 and IL1β to induce endothelial to mesenchymal transition (EndoMT) on HUVEC^40^ as this phenomenon occurs during renal IRI and is linked with fibrosis^41^ but treatment with pUPC‐EVs did not modify the EndoMT process based on gene expression levels (Figure S7).

### 3.8 Metabolomic analysis of normothermic perfusates

We performed metabolomics analysis on NMP perfusates collected at T5 using an ion trap mass analyzer with Orbitrap technology coupled with liquid chromatography and high‐resolution accurate mass spectrometry. We used a supervised analysis orthogonal partial least squares discriminant analysis to establish if the algorithm could separate the 17 perfusates, separated into three groups (Figure S8A). The results showed that samples could be clustered together, allowing us to extract the contributing variables from the orthogonal partial least squares discriminant analysis model. We then focused on the top 20 contributing metabolites (Figure S8B). Among them, we highlighted some metabolites whose presence was significantly affected by our cell therapy treatment, including (1) a decreased level of Glutathione in G2 (trend for G3) which may reflect a lower level of oxidative stress and then reduced level of this metabolite circulating within the perfusate; as well as a decrease of the withanolides‐analog signaling pathway, involved in the regulation of oxidative stress by upregulation of the Nrf2 and HO‐1 antioxidant responses; (2) a decrease of an ecdysone‐analog sharing homology to aldosterone with mineralocorticoid action^42^ that can induce mesenchymal accumulation of extracellular matrix, epithelial dedifferentiation and kidney cell apoptosis^42^, ^43^; (3) a decrease of a tolvaptan‐analog, a vasopressin receptor antagonist playing a fundamental role in water diuresis^44^; and (4) a decrease of Sulfonylureas‐analog signaling pathway which are selective inhibitors of K^+^/ATP channels playing a significant role as mediator or end effector in remote ischemic preconditioning.^45^

### 3.9 Transcriptomic analysis of kidney biopsies

**FIGURE 9:**
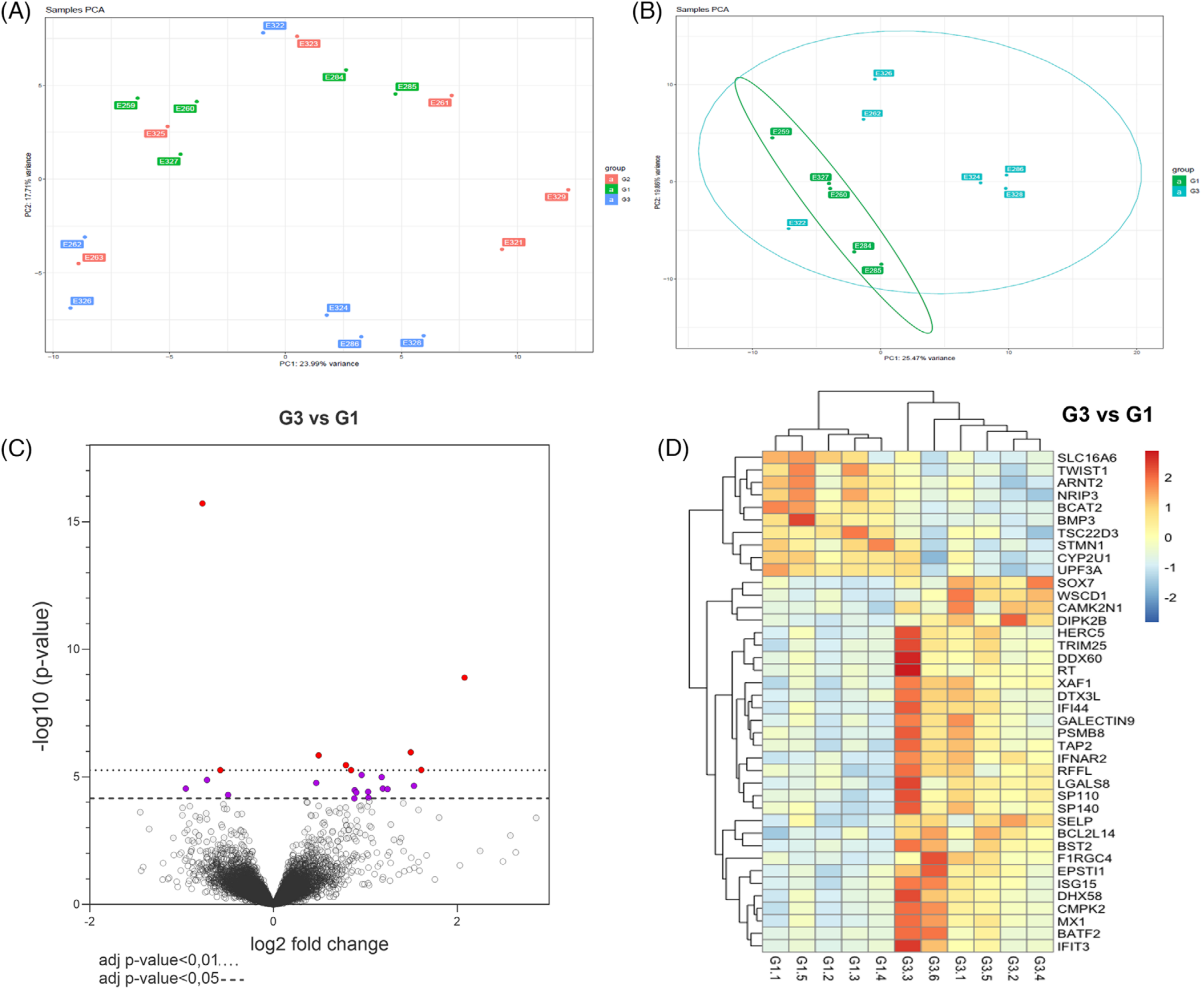
Transcriptomic analysis of kidney biopsies. (A) Principal component analysis of the RNAseq data for G1, G2, and G3 groups. (B) Principal component analysis of the RNA‐seq data for G1 and G3 groups only. (C) Volcano‐plot analysis showing differential gene expression between G1 and G3. (D) Heat map showing most differentially regulated gene expression between samples from G1 and G3; data are deposited on GEO (number GSE255005).

Finally, we performed transcriptomic analysis of kidney biopsies using RNA‐seq. PCA separated G3 samples (in blue) from G1 samples (in green), except for one outlier G1 sample. G2 samples (in red) were spread out among G1 and G3 samples (Figure 9A). For post‐transcriptomic analysis, we considered only G1 and G3 groups which were fully separated by PCA (Figure 9B). The differential gene expression pattern between G1 and G3 is represented as a volcano plot in Figure 9C. Top differentially regulated genes from the volcano plot were plotted into a heatmap (Figure 9D and Table S3) and include some genes of interest such as *SOX7, ISG15*, and *TWIST1* for instance. We then performed a functional enrichment analysis in order to highlight pathways/genes potentially involved in the observed kidney response to EVs treatment and found the following pathways affected by our cell therapy product: “immune system process”, “response to external stimulus”, “response to stress”, “regulation of response to stress”, “ISG15‐protein conjugation” notably (Figure S9). In addition, we performed a bibliographic analysis combined with functional enrichment using gprofiler on the list of pathways that were differentially expressed among G1 and G3. We quantified the expression of some selected target genes by qRT‐PCR including genes linked with ISGylation (*ISG15*), ubiquitylation (*SUMO1 and SUMO2*), cell proliferation (*PCNA*), hypoxia (*HIF1* α *)*, and immunomodulation (*SOC5* and *FOXP3*) (Figure S10).

## 4 DISCUSSION

Combining an innovative cell therapy product based on porcine UPC‐EVs with an IR ex vivo porcine kidney model, our study shows that (1) UPCs can be isolated from voided porcine urine and share a characterization profile with human UPCs; (2) pUPC‐EVs injection during HMP is feasible and does not perturb kidney perfusion parameters; (3) pUPC‐EVs injection during HMP and NMP is feasible, decreases cell damage markers and triggers an immunomodulatory profile; (4) NMP perfusates treated with pUPC‐EVs have a positive indirect impact on cultured cells in basal and hypoxic conditions; and (5) pUPC‐EVs‐treated kidneys have distinct biochemical, metabolic, and transcriptomic profiles highlighting some targets of interest.

### 4.1 The choice of the cell therapy product

The choice of UPCs among other stem cell types relies on their many advantages, among them renal origin and convenient isolation procedure. We used porcine cells to avoid hurdles linked with inter‐species approaches, thus characterizing our product was crucial (porcine UPCs were not described yet). We found that pUPCs share characteristics with their human counterparts and can differentiate into tubular epithelial cells. However, the maturity as well as functionality of the differentiated cells were not analyzed.

Testing the injection of UPCs instead of their extracellular vesicles could have been interesting. However, the scientific community has significant experience with MSC cell therapy showing that beneficial effects are due to cell paracrine effects rather than engraftment.^46^ On the other hand, one could expect that UPCs, based on their renal origin and differentiation capacity, would have additional potential to engraft and participate in renal regeneration by differentiating. We chose to test pUPC‐EVs since increasing evidence in the literature shows some benefits for stem cell‐derived EVs in different pathological contexts.^24^, ^47^, ^48^, ^49^ Moreover, EVs offer advantages compared with whole cells: (1) they may be less immunogenic despite MHC‐I expression^50^; (2) their size likely prevents them from being stuck in the perfusion circuit (or vascular bed), especially since cold storage induces vasoconstriction; (3) they can be cryopreserved at −80°C without additives. These elements may facilitate a potential transfer of this technology from bench to bedside, using EVs as intrants in preservation solutions. We attempted to follow MISEV 2023 guidelines to produce and characterize our EVs (likely belonging to the MISEV 2023 exosome category but such nomenclature would require a more exhaustive characterization) by using the gold standard Nanosight Tracking to provide a precise EVs quantification/evaluation. Our EVs expressed ALIX and TSG101, 2 recommended cytosolic proteins for exosome characterization. However, we faced issues in validating some MISEV 2023 recommended transmembrane protein expression, such as CD9 or CD81, by Western Blot due to the poor availability of pig cross‐reacting antibodies. The optimal number of EVs to inject remains a question. Few studies injected MSCs in porcine kidney models^20^, ^22^, ^51^ and it was shown that injection of up to 10 million MSCs was deemed safe.^51^ In a porcine model, previous studies showed an immunomodulatory effect of EVs derived from porcine MSCs on kidneys.^52^ They injected systemically 10 × $10^{10}$ EVs but hypothesize that only 5% of these went into the kidney, that is, 5 × $10^{9}$ vesicles with EVs representing only 10% of their injected EVs population (or 5 × $10^{8}$ EVs). We thus decided to inject the amount of EVs secreted by about 10 million UPCs, corresponding to around $.65^{10}$ pUPC‐EVs per injection.

Regarding injection timing, ischemia induces a vasoconstriction impacting kidney perfusion during the first minutes of ex vivo perfusion, thus inducing the no‐reflow phenomenon.^53^ We chose the injection timing based on a compromise between avoiding the first “chaotic” minutes of perfusion and the desire to inject them as early as possible to observe an effect. It may be further considered to repeat the injection to emphasize its effects as well as label the EVs to follow their distribution within kidneys.^52^

### 4.2 The ex vivo preservation model

We used a relevant porcine preclinical model with kidneys having anatomical, physiological, and functional similarities with their human counterparts,^54^ allowing the use of a perfusion system used in clinics and dedicated preservation solutions. Conditioning porcine grafts is also of interest in the context of xenotransplantation where recent studies brought new insights regarding genetically modified animals, immune response, and “long‐term” follow‐up after xenotransplantation.^55^, ^56^, ^57^ Our kidneys came from a collaborating slaughterhouse where animals are killed for food industry purposes, in line with the animal ethical 3R rules. The specificity of this model is the incompressible warm ischemia time (around 30 min) which impacted kidney function whatever the group; this is, however, a clinical situation occurring with organs coming from DCD donors. We chose to use a model combining both HMP and NMP; as the effect of EVs during both procedures independently was unknown and the combination of both was not described. Moreover, HMP offers a large window for extracorporal organ conditioning probably more easily applicable in clinical settings, preparing the graft for its reperfusion. We used the LifePort HMP system (Organ Recovery System) which showed benefits in clinical setting^2^ with a cold preservation time of 24 h^58^ close to the average kidney preservation time in France (Biomedicine Agency). HMP was followed by 5 h of NMP performed on a home‐made perfusion system based on previous studies including investigation on the effect of cell therapy approaches.^22^, ^59^ Indeed, one interesting idea, currently tested in preclinical and clinical studies, consists of performing a preservation sequence combining HMP and a few hours of NMP to condition the graft.^60^, ^61^, ^62^ In this context, introducing cell therapy during the NMP windows appears as a promising option.^8^

We used a relevant preclinical porcine kidney preservation model but one limitation we faced is that only short‐term analyses are feasible and the long‐term effect of our cell therapy product was not assessed here. This proof‐of‐concept study will be validated in a preclinical porcine kidney transplantation model with long‐term follow‐up of the transplanted kidney (3 months) before translating these results into human clinical trials. This model is one expertise of our laboratory^63^, ^64^ and will allow analysis of renal graft recovery, delayed graft function, graft histology (fibrosis, inflammation), etc.

### 4.3 The effect of pUPC‐EVs

Porcine UPC‐EVs did not impair kidney perfusion during either HMP or NMP, demonstrating the technical feasibility of our strategy. pUPC‐EVs did not impact kidney functional parameters (creatinine clearance and sodium excretion fraction). This might be explained by the fact that 5 h of NMP is not long enough: some parameters relative to kidney function would need to be analyzed either during a longer perfusion or after KTx analyzing both short‐term and long‐term function. Our experience in the porcine auto‐transplantation model shows that plasma creatinine reaches a peak between Days 1 and 3 post‐Tx.^63^

We observed that pUPC‐EVs injection performed in both HMP and NMP: (1) decreases cell membrane damage/necrosis (LSH, ASAT) markers released; (2) reduces circulating stress‐responsive markers (HO‐1, IL‐18) and pro‐inflammatory cytokines (IL‐1α, IL‐1β). ASAT and LDH are widely used as markers for necrosis, as they are released during cell death. Moreover, ASAT is also used as a biomarker in kidney IR.^65^ Here, we observed a decrease in ASAT and LDH levels in G3 compared with G1 (AUC analysis); however, these results should be considered with caution since kidneys from G3 already seemed to have lower LDH/Lactate levels at the time of EVs injection (no performed statistics). Concerning IL‐18, the decrease in G3 is considered a sign of diminished stress injury,^66^ similar to the decrease of HO‐1, a stress‐responsive enzyme.^67^ Overall, our results seem in favour of an immunoregulatory balance in G3. This finding, in line with the literature, shows the immunomodulatory properties of EVs from MSCs^68^ and USCs.^32^ We also observed a decreased expression of IGF1, VEGFA, and ANG in G3. These results may suggest that EVs injection would limit renal damage during reperfusion, thus limiting the need for kidneys to secrete those factors involved in cellular repair. However, due to the experimental design, the exact cytokines’ origin cannot be precisely established: either initially present in the EVs or secreted by the kidney in response to the treatment. Regarding metabolism, the important lactate production/accumulation might reflect the metabolic shift occurring when the tissue faces hypoxic stress. Lactate level is decreased in G3 at the oxygenated reperfusion time in NMP; this may be linked to a better oxidative metabolism recovery. This result is concordant with our in vitro data suggesting a better preservation of mitochondrial activity. Finally, our results from histological analysis seemed to show that kidneys from G3 displayed a better‐preserved structure, compared with kidneys from G1 and G2 with reduced tubular dilatation (tubular flattening), cell detachment, and inflammation. However, kidney biopsies were performed after only 5 h of normothermic perfusion, limiting our interpretation and must be addressed further in a long‐term model of reperfusion. Moreover, CD10 staining, reflecting the integrity of the brush border, was enhanced in G3. Finally, we observed an increased staining of Vimentin in G3, which could be explained as Vimentin expression is required during the early phases of renal reparation.^69^ Importantly, those results should be further validated in a long‐term transplantation model which is necessary to assess the impact of the observed changes (at the cellular and tissular level) on the graft function.

### 4.4 Toward deciphering mechanism of EVs action

We took advantage of a model producing well‐controlled perfusates and tissues to decipher the mechanisms underlying the observed benefits using in vitro assays, metabolomics, and transcriptomics. First, perfusate containing EVs directly increased the metabolic activity of endothelial cells in basal and hypoxia‐reoxygenation conditions. Very recent studies provided significant insights into the proteome/metabolome of kidneys preserved with HMP.^70^, ^71^ Second, we showed the possibility of performing open metabolomic analysis on perfusion liquids collected from kidneys undergoing normothermic perfusion machine. Indeed, using an ion trap mass analyzer with Orbitrap technology coupled with LC/MS, a supervised classification led to the identification of the Top20 metabolites discriminating against our samples. Among those, we highlighted some notably linked with oxidative stress (glutathione, withanolides), homeostasis maintenance (tolvaptan, ecdysone), and transporters. While the pathways uncovered are consistent with the ischemia‐reperfusion mechanism, these metabolomics investigations require further experiments, including in vivo testing; however, those results demonstrate the feasibility and pertinence of metabolomics investigation of the perfusate, herein showing the in‐depth effects of our therapeutic approach and a possible source of effectiveness biomarkers. Finally, we performed transcriptomic analyses on biopsies collected after the NMP procedure. RNA‐seq data correctly discriminated our samples by PCA. We highlighted several genes that were differentially expressed between G1 and G3 which may participate in pUPC‐EVs action mechanism. Heatmap representation showed that the most regulated gene expression pattern was consistent among the different samples of our groups. Moreover, functional enrichment analysis revealed some involved pathways (Figure S9) that deserve further studies; nevertheless, using a complementary bibliographic‐biased approach the expression fold change was quantified for some of our target genes confirming that our cell therapy product induces changes in pathways linked with response to stress and hypoxia, cell proliferation, immunomodulation, and post‐translational modifications notably. Finally, one strategy to decipher the mechanism of action of our cell therapy product would have been to assess the EVs cargo by proteomic analysis notably, which is planned in a further study, as well as reproducing these results with EVs from cells coming from different donors to validate our cell therapy product.

## 5 CONCLUSION

Altogether, this study brings the proof of concept of the feasibility, safety, efficacy (diminution of cell damage markers, immunomodulation, better kidney structure integrity), and putative mechanisms (metabolomics and transcriptomic analyses) of complementing ex vivo kidney dynamic preservation solution with a cell therapy product based on urine stem cell‐derived EVs to improve its condition. Our promising results pave the way for combining machine perfusion with EVs‐based cell therapy for organ conditioning.

## AUTHOR CONTRIBUTIONS

Conceptualization: Perrine Burdeyron, Sébastien Giraud, Henri Leuvenink, Luc Pellerin, Thierry Hauet, and Clara Steichen. Methodology: Perrine Burdeyron, Sébastien Giraud, Raphaël Thuillier, Henri Leuvenink, Luc Pellerin, Thierry Hauet, Clara Steichen. Investigation: Perrine Burdeyron, Sébastien Giraud, Maryne Lepoittevin, Sonia Brishoual, Maïté Jacquard, Virginie Ameteau, Nadège Boildieu, Estelle Lemarie, Jonathan Daniel, Frédéric Martins, Nicolas Mélis, Marine Coué, Raphaël Thuillier, Henri Leuvenink, Luc Pellerin, Thierry Hauet, Clara Steichen. Visualization: Perrine Burdeyron, Sébastien Giraud, Nina JordanThierry Hauet, and Clara Steichen. Supervision: Sébastien Giraud, Thierry Hauet, Clara Steichen. Writing—original draft: Perrine Burdeyron, Sébastien Giraud, Thierry Hauet, Clara Steichen. Writing—review & editing: Perrine Burdeyron, Sébastien Giraud, Nicolas Mélis, Thierry Hauet, and Clara Steichen.

## CONFLICT OF INTEREST STATEMENT

The authors declare no conflict of interest.

## ETHICAL APPROVAL

Approval was obtained from the local ethics committee.

## CONSENT FOR PUBLICATION

Not applicable

## SUPPORTING INFORMATION STATEMENT

Additional supporting information may be found online in the Supporting Information section.

## Supporting information



Supporting Information

Supporting Information

## Data Availability

All data are presented in the article except for transcriptomic data which are deposited on GEO (number GSE255005).
