# Supplementary material for: Dynamic conditioning of porcine kidney grafts with extracellular vesicles derived from urine progenitor cells: A proof‐of‐concept study
Source: Clin Transl Med. 2024 Dec 13;14(12):e70095. doi: 10.1002/ctm2.70095 (PMC11645449; doi:10.1002/ctm2.70095)
Supplement: Supplementary file 1 — Supporting Information [file CTM2-14-e70095-s002.docx]

Supplementary Materials for:

**Dynamic conditioning of porcine kidney grafts with exosomes derived from Urine Progenitor Cells: a proof of concept study**

Burdeyron et al.

Fig. S1.: Detailed summary of pUPC characterization. The isolation procedure was performed for three different urine samples and led to the isolation of pUPC colonies that we amplified for cryopreservation and characterization; a. Results for 3 different pUPC lines: b. Studied protein and target and used positive/negative control are indicated in columns. Expression results are expressed as positive (> 80% of the cells, in green) or negative (<5% of the cells, in red) in the adequate column depending on the used technique. All these characterization tests were performed for the pUPC3 cells.


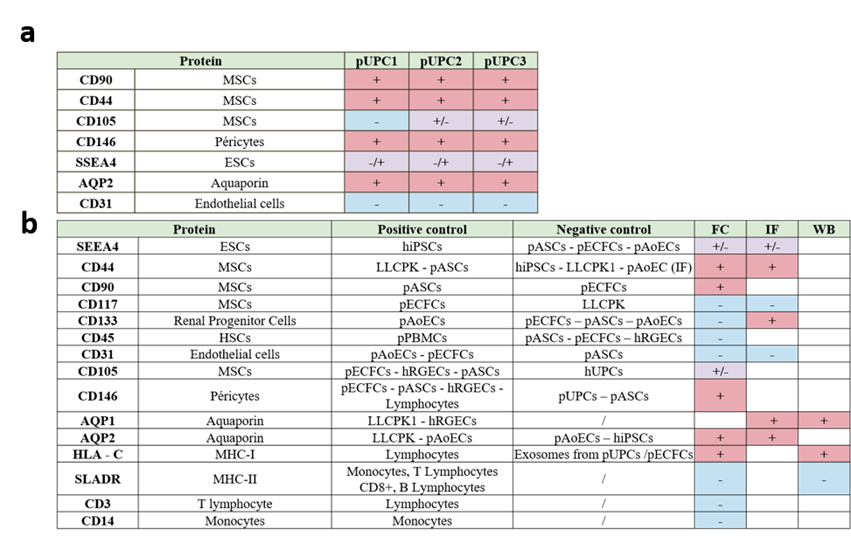


Fig. S2.: pUPC differentiation into epithelial cells: a: Cell morphology during differentiation at Day 0, Day 8 and Day 21 of differentiation; b: Analysis of differential gene expression in differentiated pUPC (D21) compared to D0 for E-cadherin, Aquaporin 3, Vimentin, αSMA (Smooth Muscle Actin) and VCAM1 (fold changes calculations are detailed in the Material and Methods section); c: Western Blotting showing the protein expression pattern of AQP-1 at D21 compared to D0 as well as its glycosylation pattern (upper image); quantified repartition of the different forms of the protein is represented in the graph on the lower panel. D0 and D0bis are two different differentiation experiments, leading to D21 and D21bis samples.


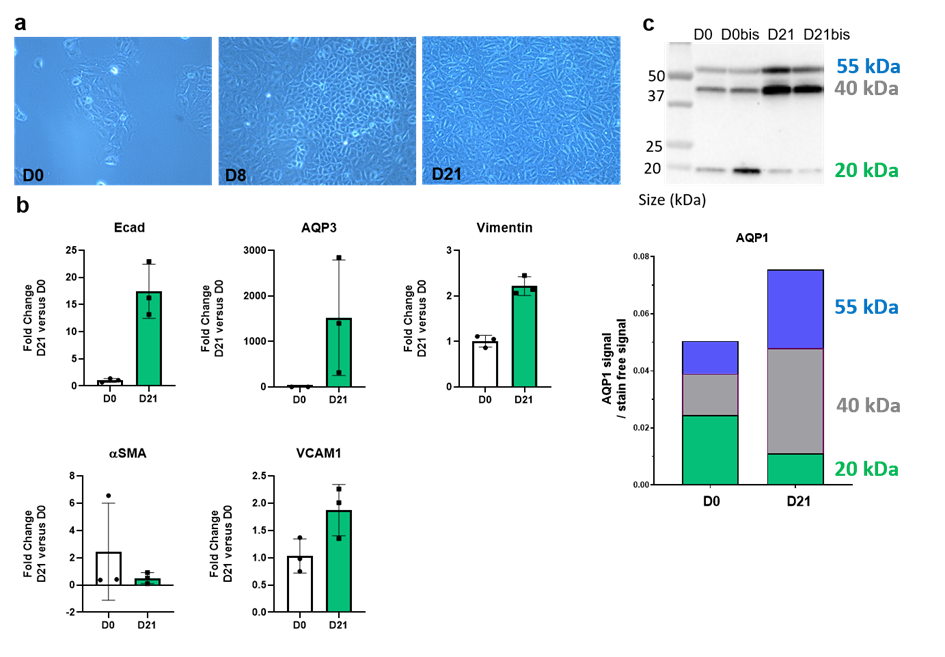


Fig. S3.: Western Blotting for ALIX and TSG101 in pUPC-exosomes: a: Analysis of TSG101 by Western Blotting for: Exosomes derived from pECFCs (porcine endothelial colony forming cells), exosomes derived from pUPCs, pECFCs (cell lysate), pUPCs (cell lysate), exosomes derived from whole urine used as positive control; upper line: stain-free technology representing the total amount of proteins (same blot) b: Analysis of ALIX by Western Blotting for: Exosomes derived from pECFCs (porcine endothelial colony forming cells), exosomes derived from pUPCs, pECFCs (cell lysate), pUPCs (cell lysate), exosomes derived from whole urine used as positive control; upper line: stain-free technology representing the total amount of proteins (same blot).


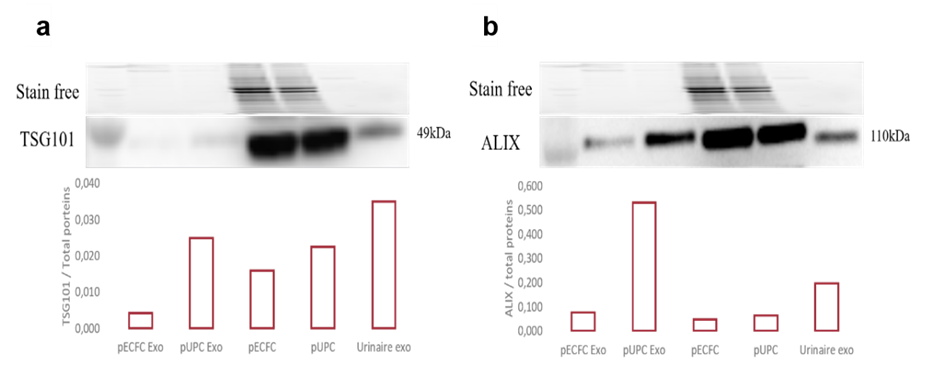


Fig. S4.: Immunostaining score of kidney biopsies with Vimentin: Meaning for statistical differences : Within each group, T0 versus T5 : * p<0.05, ** p<0.01, *** p<0.001 (Mann Whitney Test); Between groups, T0 vs T0 :  § p<0.05, §§ p<0.01, §§§ p<0.001 (Kruskal-Wallis + Dunn's multiple comparisons test), # p<0.05, ## p<0.01, ### p<0.001 (Kruskal-Wallis + Dunn's multiple comparisons test); V+ c: Vimentin-positive cells

**
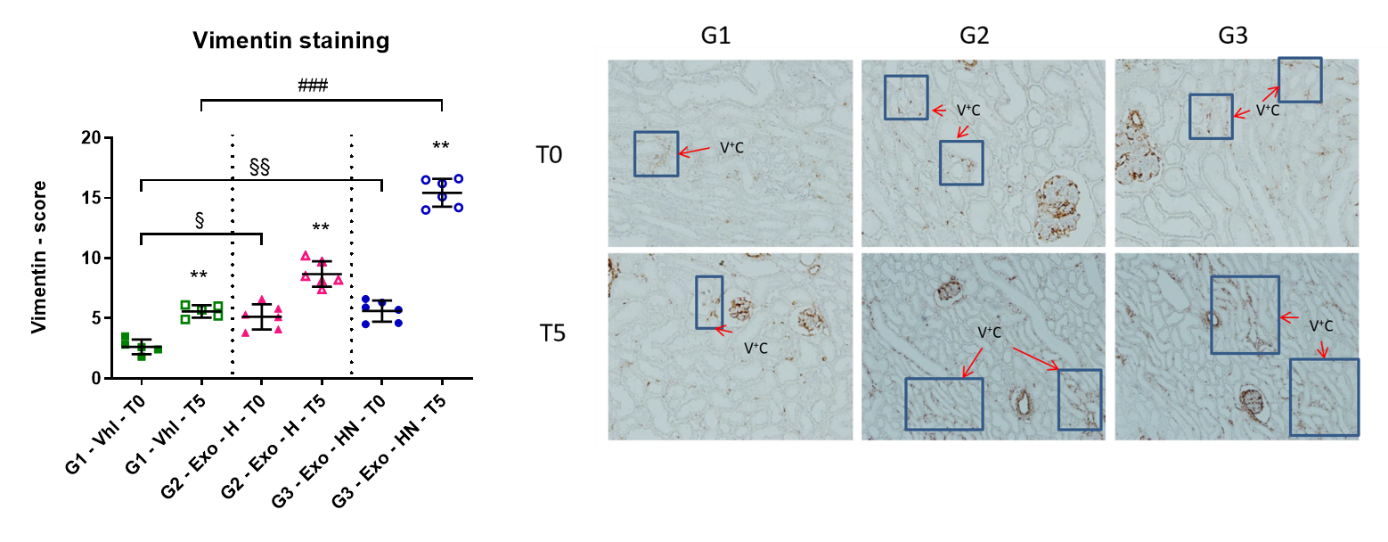
**

**Fig. S5.:** HMP liquid analysis: Kinetics (left panel) and AUC (right panel) analyses of perfusates gathered during the HMP procedure (For IL18, *P* = 0.037 (G3 vs G1) and 0.025 (G3 vs G2)). No graph was performed for HO1 analysis since this protein was detected only in perfusates from G1.


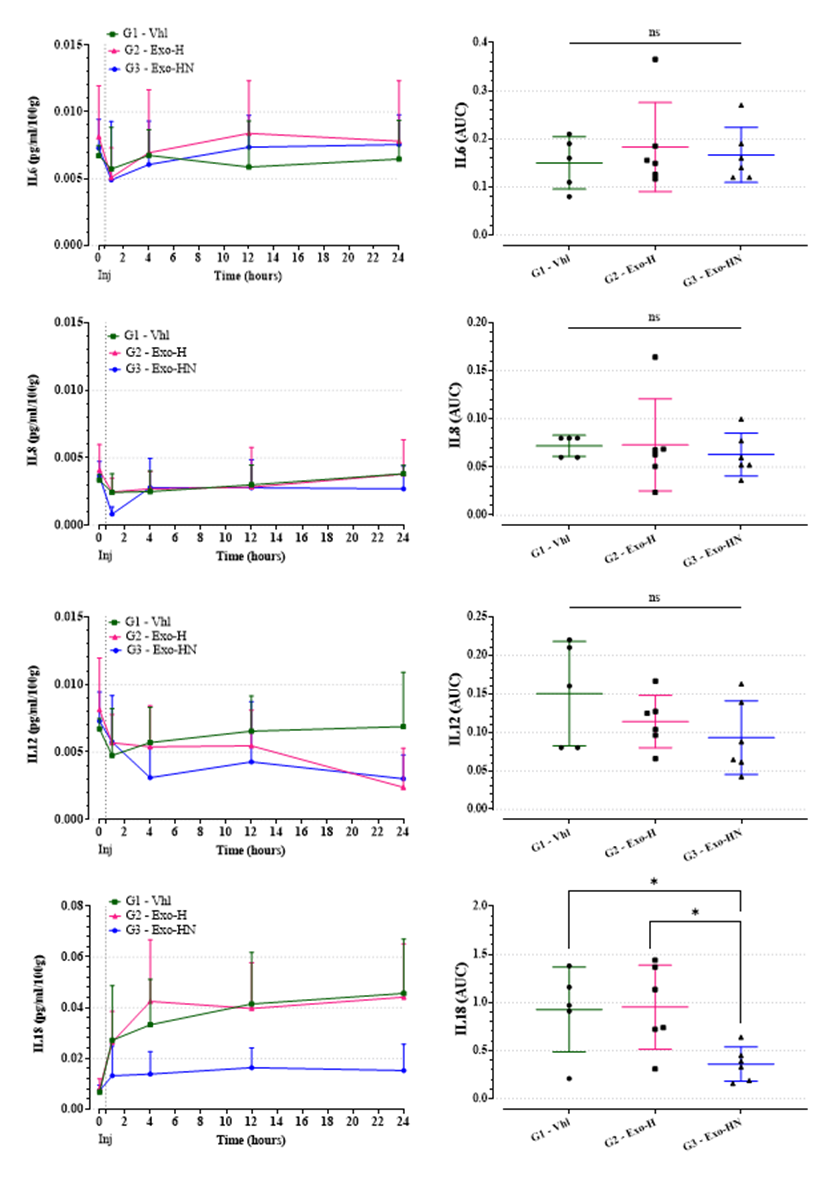


**Fig. S6.:** NMP liquid analysis: Kinetics (left panel) and AUC (right panel) analyses of perfusates gathered during the NMP procedure.


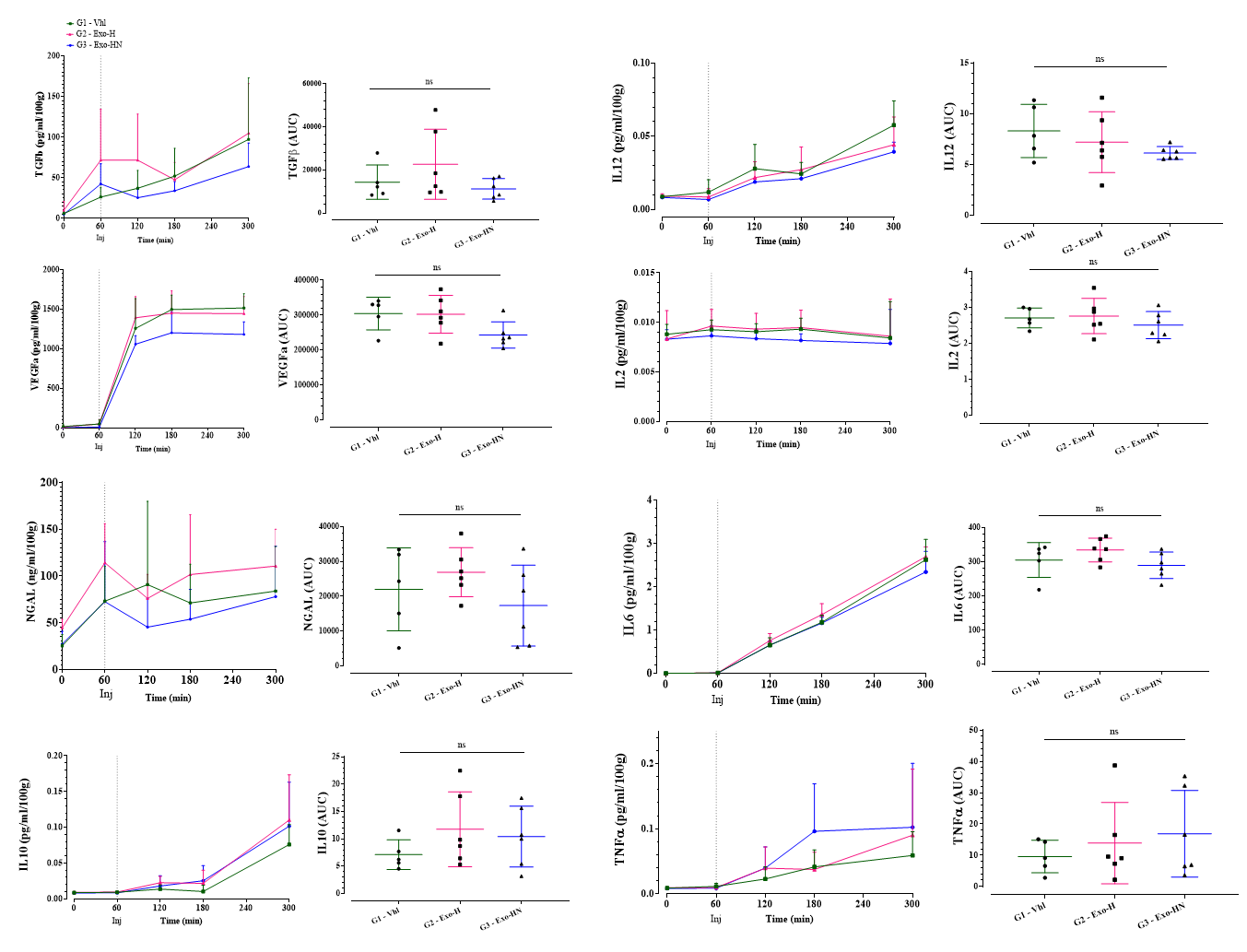


**Fig. S7.:** Endothelial to mesenchymal transition (EndMT) *in vitro:* Exosomes isolated from UPCs do not prevent endothelial-to-mesenchymal transition *in vitro*: HUVEC cells were treated with TGFβ2 and IL1β (10ng/ml) to induce EndMT as previously described. Exosomes isolated from UPCs were incubated with the cells undergoing endothelial-to-mesenchymal transition. Gene expression of EndMT markers such as SNAI1, CD31 and VWF were analyzed 48 and 72h post-treatment. Data from cells post-treatment were normalized to data from cells without treatment. (N=3)


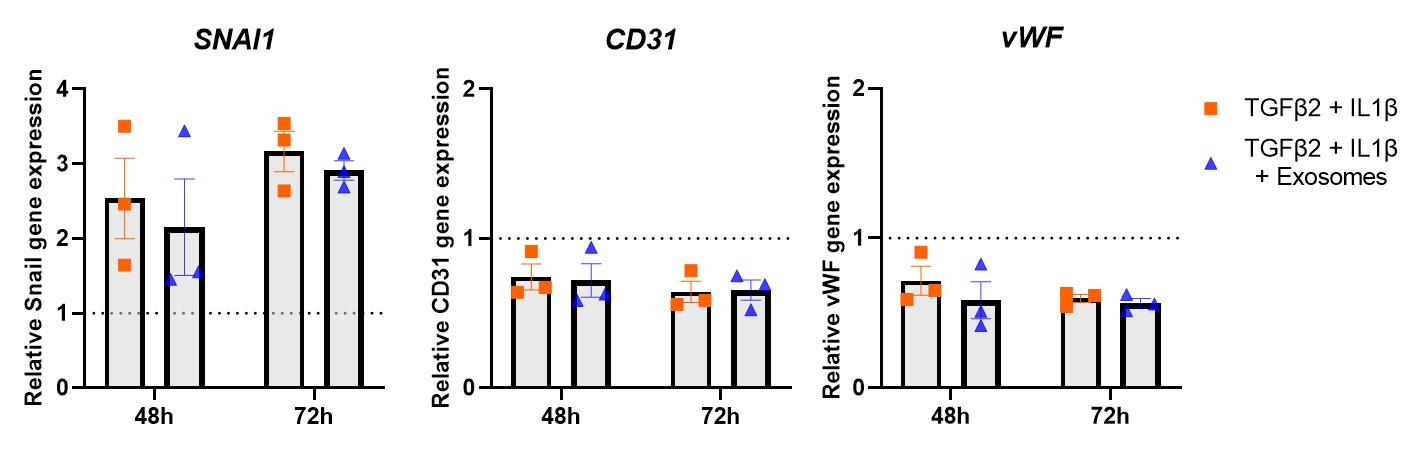


**Fig. S8.:** Top-20 metabolites following NMP perfusate analysis with LC/MS


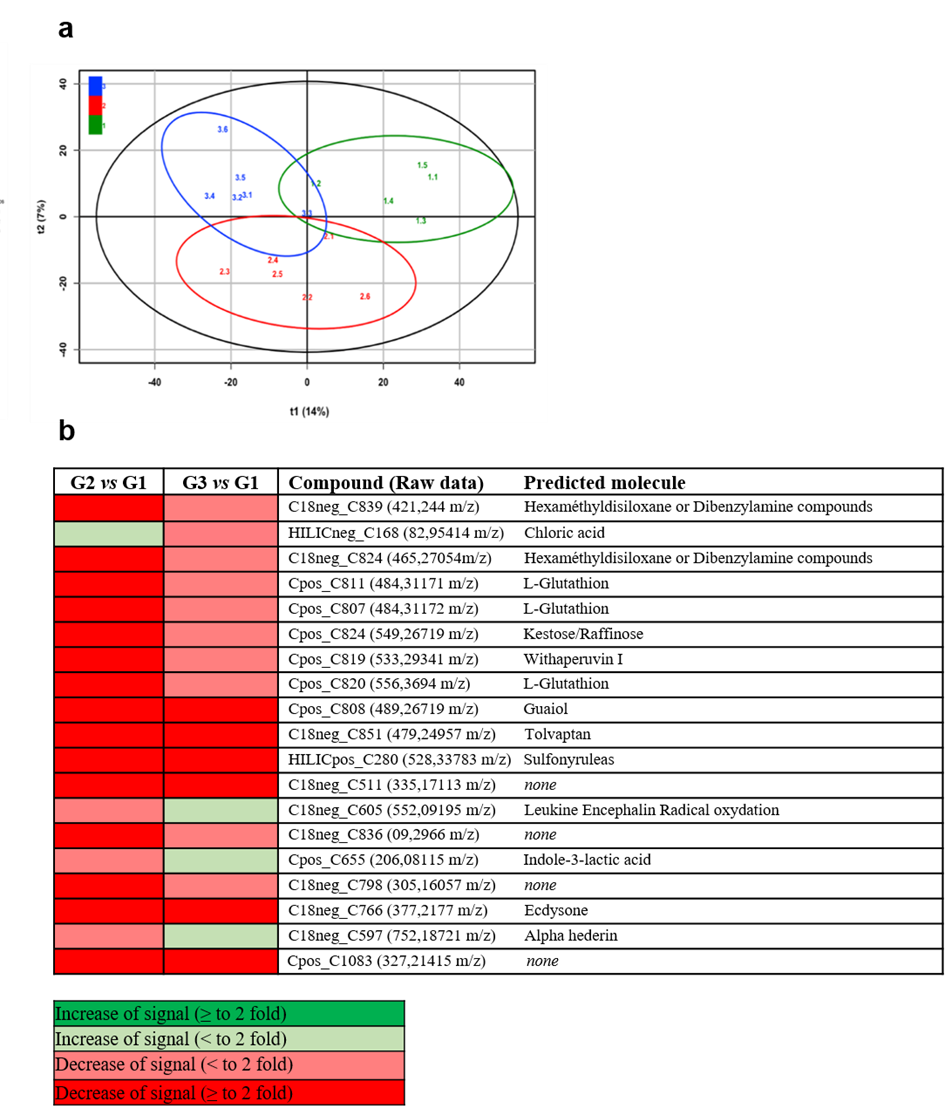


**Fig. S9.:** Functional enrichment analysis was performed with g-profiler web platform (<https://biit.cs.ut.ee/gprofiler/gost>) with the most statistic genes regulated (p<0.05) from the RNA-seq data. Functional enrichment analysis wrere performed by REACTOME pathway database (REAC; first figure), and by Gene Ontology(GO) to obtain two subontologies: GO Molecular Functions (GO:MF; second figure) that describe gene product's functions, and GO Biological Process (GO:BP; third figure) that describe in which biological process the gene product participates.

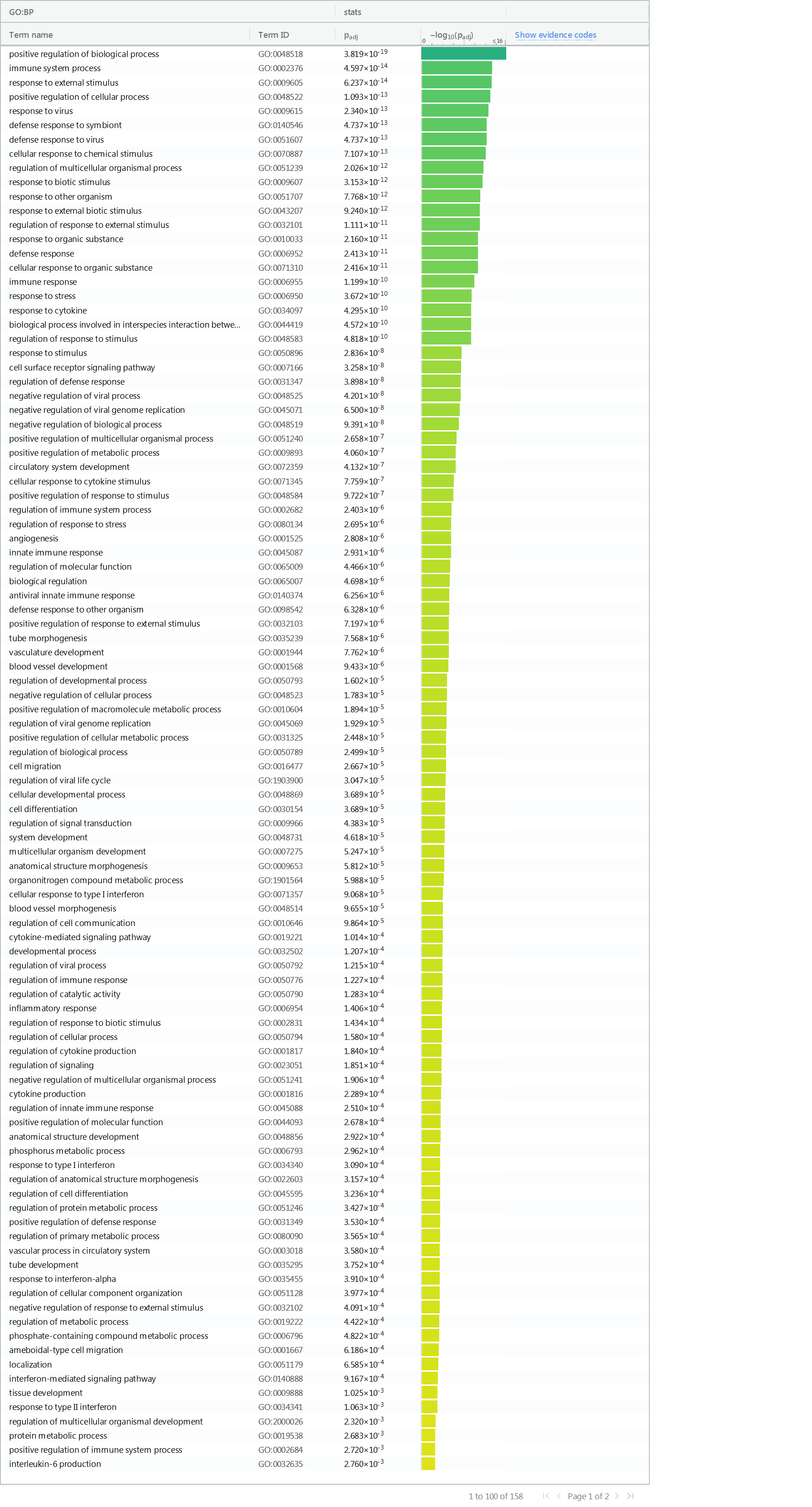


**Fig. S10.:** qRT-PCR on some target genes from RNAseq data


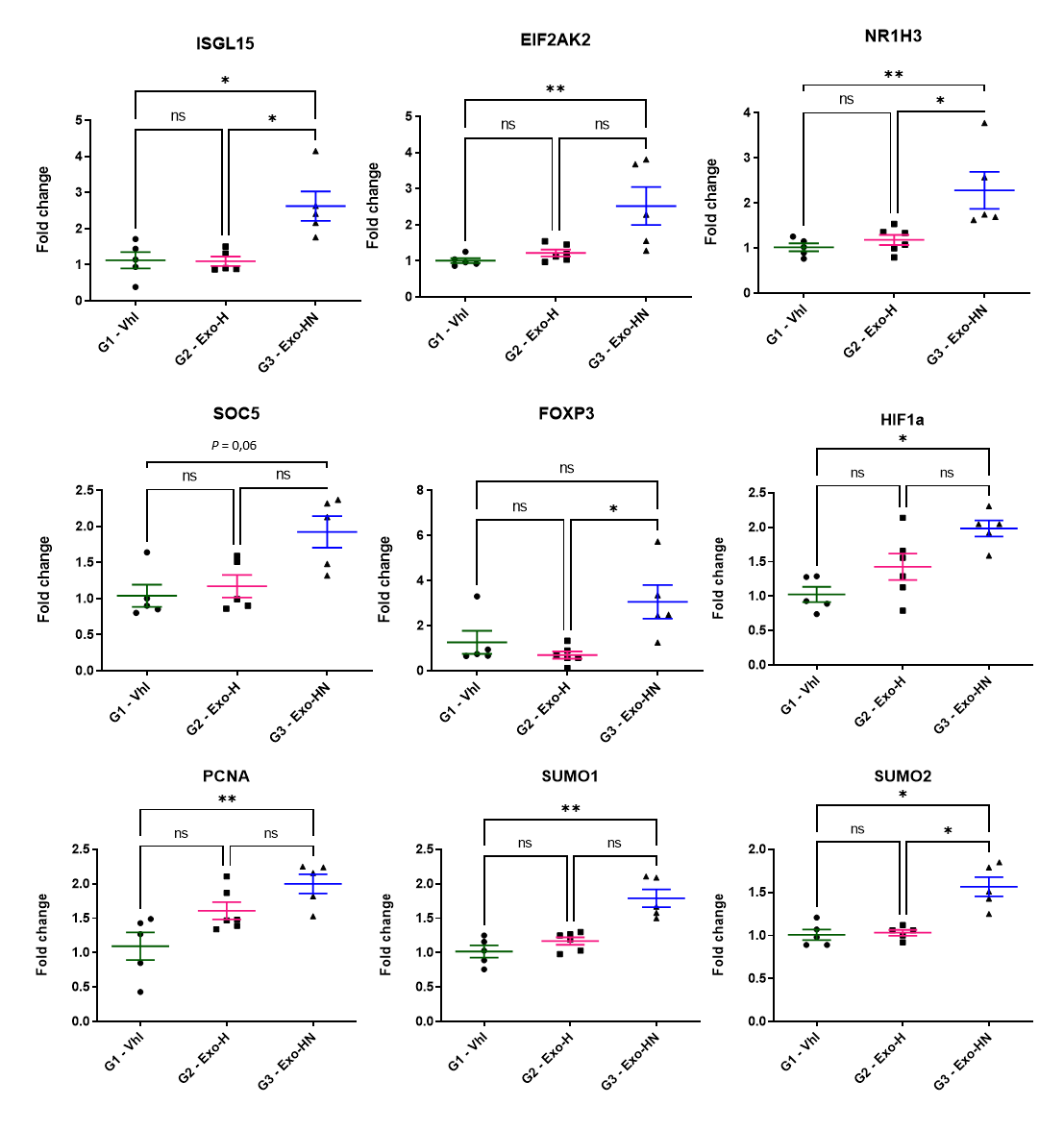


Table S1.: antibodies used for immunofluorescence, western blotting and flow cytometry

| **Protein** | **References** | **Dilution** | **Isotype or secondary antibody** | **References** | **Dilution** |
| --- | --- | --- | --- | --- | --- |
| **AQP1** | Biorbyt, orb10122 | 1/100 FC, | goat to rabbit alexa 488 | Invitrogen A32731 | 1/500 FC |
| **AQP2** | Biorbyt orb235021 | 1/300 FC et IF | goat to rabbit alexa 488 | Invitrogen A32731 | 1/500 FC |
| **CD3** | Abcam, ab25413 | 1/100 FC | IGg1-PE | Invitrogen, MG104 | 1/100 FC |
| **CD14** | AbD serotec, MCA1218F | 1/50 FC | IgG2b - FITC | Invitrogen, MG2B01 | 1/100 FC |
| **CD31** | Biorad, #MCA1746PE | 1/300 IF | Goat to mouse alexa 488 | Abcam ab150113 | 1/1000 IF |
| **CD31** | Ab Serotec, MCA1746F | 1/10 FC | IgG1 - FITC | Invitrogen, MG4992 | 1/100 FC |
| **CD44** | Biorad MCA1449GA | 1/100 FC et IF | Goat to rat Alexa 488 | Abcam ab150157 | 1/500 FC, 1/1000 IF |
| **CD45** | Biorad, MCA1222D680GA | 1/50 FC | IGg1 647 | Invitrogen, MG121 | 1/100 FC |
| **CD90** | Abcam, ab139364 | 1/50 FC | IgG1 APC | Invitrogen, MG105 | 1/100 FC |
| **CD105** | Novus biological NB100-65601-PE | 1/50 FC | IgG2a-PE | Invitrogen MG2A04 | 1/100 FC |
| **CD117** | Biorad MCA2598GA | 1/500 IF | Goat to mouse alexa 488 | Abcam ab150113 | 1/1000 IF |
| **CD133** | Abcam, ab19898 et Novus Biological NB 120-16518 | 1/100 FC, 1/500 | goat to rabbit | Abcam ab6721 | 1/500 WB |
| **CD146** | Serotec, MCA2141A647 | 1/50 FC | IgG1 647 | Invitrogen, MG121 | 1/100 FC |
| **SEEA4** | Abcam, ab16287 | 1/300 FC, IF et WB | Goat to mouse alexa 488 | Abcam ab150113 | 1/500 FC |
| **HLA-C** | Briorad, MCA2261F | 1/10 FC | Goat to mouse alexa 488 | Abcam ab150113 | 1/100 FC |
| **SLADR** | BD Bioscience, 553642 | 1/100 FC | Goat to mouse alexa 488 | Abcam ab150113 | 1/500 FC |

Table S2.: primer sequences

| **Gene** | **Sequence (5’ 🡪 3’)** |
| --- | --- |
| αSMA – Forward | GGGGTGATGGTGGGAAT |
| αSMA – Reverse | TGCCGTGTTCTATTGGGTACT |
| AQP3 – Forward | ACCACGTGGAAAGGACTCTAGC |
| AQP3 – Reverse | TTTCCTCACACGTGGACACACC |
| CYA62 – Forward | AAGACTGAGTGGTTGGATGG |
| CYA62 – Reverse | AATGGTGATCTTCTTGCTGGT |
| ECAD – Forward | GTATCGGATTTGGAGGGATG |
| ECAD – Reverse | TCGGCCCGAGTGGTAAT |
| VCAM1 – Forward | ATTTCTCCGGATGGTACTGCC |
| VCAM1 – Reverse | AAAGTTACACAAGAGTCTGATGAACAAAC |
| VIMENTIN – Forward | AGGTGGACCAGCTCACCAA |
| VIMENTIN – Reverse | ATGATGTCCTCGGCCAGATT |
| pISG15 – Forward | ATGATGGCATCGGACCTGAA |
| pISG15 – Reverse | TGTCGTTCCTCACCAGGATG |
| pSOCS5 – Forward | CTGTGTTTGCAGTCTCGGAG |
| pSOCS5 – Reverse | GCTTCGTAACGGTCCATCAC |
| pFOXP3 – Forward | GTGACAGTTTCCCACAAGCC |
| pFOXP3 – Reverse | GGGGTTCAAGGAGGAAGAGG |
| pEIF2AK2 – Forward | CCATCTGGTCATCATATATCCCACT |
| pEIF2AK2 – Reverse | AGAAACACGGTGAACGACC |
| pNR1H3 – Forward | CAGCTCACCTCACAGAAGAGT |
| pNR1H3 – Reverse | CCACCCACAAGGACATCTCT |
| pSUMO1 - Forward | TCAACTGAGGACTTGGGTGA |
| pSUMO1 – Reverse | CTAAACCGTTGAGTGACCCC |
| pSUMO2 – Forward | GACGAAAAGCCCAAGGAAGG |
| pSUMO2 – Reverse | GTCTGCTGCTGGAACACATC |
| \| pHIF1a –Forward \|  \| \| --- \| --- \| \|  \|  \| | GCCTCTGAAACTCCAAAGCC |
| pHIF1a – Reverse | TCTTGAATCTGGGGCATGGT |
| pPCNA - Forward | AGCCACTCCACTCTCTCCTA |
| pPCNA – Reverse | GCATCACCGAAGCAGTTCTC |

**Table S3. Top-regulated genes in G3 vs G1:** P-values were determined with a Wald test and were adjusted with Benjamini and Hochberg procedure.

| **Gene** | **Gene_ID** | **Log2 Fold Change** | **p-adjusted value** |
| --- | --- | --- | --- |
| **ACSL5** | ENSSSCG00000031789 | 0,55999345 | 0,050992 |
| **ARRDC4** | ENSSSCG00000002252 | 0,738540681 | 0,049722 |
| **BCAT2** | ENSSSCG00000003139 | -0,762059238 | 0,00000001 |
| **BST2** | ENSSSCG00000033453 | 1,966048037 | 0,000093 |
| **CCL16** | ENSSSCG00000040940 | 1,509816507 | 0,010227 |
| **CMPK2** | ENSSSCG00000008647 | 1,136450967 | 0,059331 |
| **CYP2U1** | ENSSSCG00000009151 | -0,57786892 | 0,003211 |
| **CYP4A23** | ENSSSCG00000003891 | 3,918873348 | 0,004434 |
| **DBP** | ENSSSCG00000003148 | -1,386984107 | 0,053886 |
| **DDX60** | ENSSSCG00000009720 | 1,1883255 | 0,044869 |
| **DIPK2B** | ENSSSCG00000026984 | 1,648198158 | 0,000009 |
| **ENTREP1** | ENSSSCG00000005249 | 1,088684687 | 0,003211 |
| **ERG** | ENSSSCG00000022318 | 0,537848874 | 0,001572 |
| **GC** | ENSSSCG00000027609 | 1,793944419 | 0,016078 |
| **GLB1L2** | ENSSSCG00000015258 | 0,729061988 | 0,046329 |
| **GREB1** | ENSSSCG00000025483 | -2,277424278 | 0,027242 |
| **IFI44** | ENSSSCG00000003763 | 0,860955747 | 0,024780 |
| **IFIT3** | ENSSSCG00000061268 | 1,322728496 | 0,020866 |
| **IFITM1** | ENSSSCG00000060622 | 0,8410993 | 0,000299 |
| **INHBB** | ENSSSCG00000038610 | 0,95626728 | 0,000006 |
| **LIMCH1** | ENSSSCG00000008799 | -0,811863642 | 0,050992 |
| **MEGF6** | ENSSSCG00000024678 | -1,031189202 | 0,022286 |
| **MX1** | ENSSSCG00000012077 | 1,082728878 | 0,055481 |
| **NRIP3** | ENSSSCG00000014570 | -0,738026862 | 0,026538 |
| **NT5DC1** | ENSSSCG00000004435 | -0,49990471 | 0,029110 |
| **PER3** | ENSSSCG00000028572 | -0,635525384 | 0,008097 |
| **POLH** | ENSSSCG00000001683 | -0,703325897 | 0,005330 |
| **PSMB8** | ENSSSCG00000026951 | 0,773896697 | 0,055270 |
| **SAMD11** | ENSSSCG00000033509 | -1,041507328 | 0,055636 |
| **SLC16A6** | ENSSSCG00000017262 | -1,037595391 | 0,005979 |
| **SOX7** | ENSSSCG00000031889 | 0,914245659 | 0,001222 |
| **SP110** | ENSSSCG00000016261 | 0,449194688 | 0,007111 |
| **STMN1** | ENSSSCG00000026257 | -0,573065176 | 0,055270 |
| **TAP2** | ENSSSCG00000025593 | 0,725343788 | 0,014174 |
| **TCF4** | ENSSSCG00000004535 | 0,622043138 | 0,011191 |
| **TEF** | ENSSSCG00000035152 | -1,182711844 | 0,000236 |
| **TKFC** | ENSSSCG00000013087 | -0,58829693 | 0,053990 |
| **UPF3A** | ENSSSCG00000024679 | -0,651107527 | 0,000043 |
| **WSCD1** | ENSSSCG00000017894 | 1,194811275 | 0,00000001 |
| **XAF1** | ENSSSCG00000056284 | 1,111557216 | 0,036749 |
